# A hypomorphic *Mpi* mutation unlocks an in vivo tool for studying global N-glycosylation deficiency

**DOI:** 10.1172/jci.insight.180752

**Published:** 2025-07-22

**Authors:** Elisa B. Lin, Steve Meregini, Zhao Zhang, Avishek Roy, Tandav Argula, James M. Mitchell, William J. Israelsen, Sara Ludwig, Jamie Russell, Jiexia Quan, Sara Hildebrand, Evan Nair-Gill, Bruce Beutler, Jeffrey A. SoRelle

**Affiliations:** 1 Division of Genomic and Molecular Pathology, Department of Pathology;; 2 Division of Endocrinology, Department of Internal Medicine;; 3 Center for Genetics of Host Defense;; 4 Department of Biochemistry; and; 5 Division of Allergy/Immunology, Department of Pediatrics, University of Texas Southwestern (UTSW) Medical Center, Dallas, Texas, USA.

**Keywords:** Gastroenterology, Genetics, Genetic diseases, Glycobiology, Mouse models

## Abstract

Glycans are one of the 4 major macromolecules essential for life and are the most abundant family of organic molecules. However, in contrast with DNA and RNA, glycan structures have no template; this results in limited tools to study this challenging macromolecule with a diversity of glycan structures. A central bottleneck in studying glycosylation in vivo is that inhibitors and complete KOs are lethal. In a forward genetic screen, we identified a viable, hypomorphic mutation at a conserved site in mannose phosphate isomerase (*Mpi*) that causes a multisystemic phenotype affecting RBCs, liver, stomach, intestines, skin, size, fat, and fluid balance in mice. The phenotype could be rescued with mannose. Analyses of glycopeptides in mice with this mutation showed a 500% increase in unoccupied N-glycan sites. This is equivalent to a “glycan knockdown,” which would be useful for examining the role of glycans in biology and disease. Therefore, we report an in vivo tool to study global N-glycosylation deficiency with tissue-specific targeting and a rescue mechanism with mannose.

## Abstract 2

A hypomorphic Mpi mutation mimics an inborn glycosylation disorder, enabling future in vivo studies that require global N-glycosylation deficiency.

## Graphical abstract

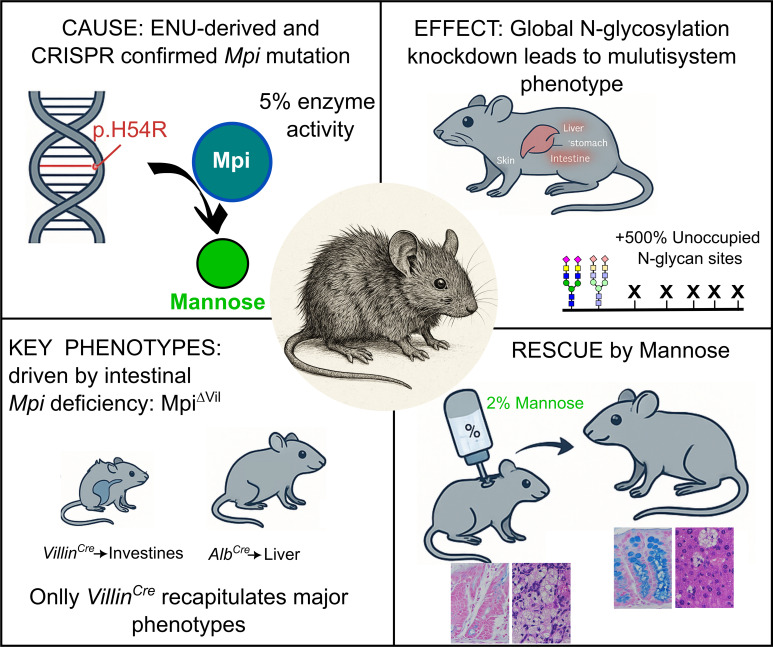

## Introduction

As many as 50% of human proteins are reported to be glycosylated (1). This posttranslational modification predominates in surface and secreted proteins, which are involved in many aspects of physiology; therefore, perturbations in glycosylation affect many physiologic and disease processes, including inflammation, cancer, immunoglobulin therapeutics, and cardiovascular disease (2–6). Glycans are added to proteins by O- and N-linked glycosylation in the ER and Golgi apparatus. Congenital disorders of glycosylation (CDGs) have provided significant mechanistic insights into systemic, physiologic roles performed by glycans (7).

Tools for studying glycobiology are limited to lectin analyses, mass spectrometry, and glycan arrays, with very few mouse models available. In vivo models are challenging, as type 1 CDG gene KOs are embryonic lethal (e.g., mannose phosphate isomerase [*Mpi*], phosphomannomutase 2 [*Pmm2*]) (8–11). Attempts to create a KO *Mpi* mouse have caused lethality; however, knocking in a hypomorphic human mutation (15% residual activity, p.Y255C) (8, 12) caused no phenotype. Just as the gold standard to assess gene function is to knock out that gene, the gold standard to assess glycan function is to mutate a glycan site. Since performing site-specific mutagenesis is infeasible in vivo, genetically turning off *N*-glycosylation is an attractive alternative. Thus, exploring the critical impact of glycosylation has been limited to in vitro settings because of a paucity of animal models where physiologically relevant insights can be made.

*MPI* deficiency in humans was one of the first identified disorders (CDG-1b = MPI-CDG); it is extremely rare (<1:1,000,000) (13) and characterized by protein-losing enteropathy (PLE), chronic diarrhea, cirrhosis, cyclic vomiting, anemia, and hyperinsulinemic hypoglycemia (14, 15). This deficiency is unique, as it is the only CDG with a known treatment, which is mannose supplementation (16). Similarly, it has been found that mannose supplementation rescued mice with deficiency in PMM2 (analogous to CDG-1a) from embryonic lethality (17). Another study found that fucokinase deficiency–related CDG may also respond to mannose therapy (18). It also appears that there is little toxicity seen in fully grown and nonpregnant mice on mannose therapy, and that levels of mannose as high as 20% can be tolerated (19). MPI is the rate-limiting step in mannose synthesis and is essential for N-linked glycosylation (20). MPI interconverts fructose-6-phosphate (from the glycolysis metabolic pathway) to mannose-6-phosphate (M6P). This is the predominant pathway for mannose synthesis, which is critical for the core structure in N-linked glycosylation (20). Exogenous mannose bypasses the MPI enzyme and allows rescue experiments (15, 20).

In a forward genetic screen in mice (21, 22), an *N*-ethyl-*N*-nitrosourea–induced (ENU-induced) point mutation in *Mpi* (p.H54R) was found that maps to an immune phenotype named *benadryl*. This allele was reproduced by CRISPR knockin. Although the immune phenotype is being investigated separately (JAS, UTSW, Dallas, Texas, USA, unpublished observations), we have compared the murine phenotypes to the characteristics of human disease (anemia, cirrhosis, low protein edema, low BW, intestinal disease, coagulopathy, and cure by supplemental mannose). Considering the ubiquitous expression of N-glycosylation, we anticipate other organ systems will be affected, but our efforts primarily focused on demonstrating parallel phenotypes compared to the human disease. Furthermore, we created *Mpi*–conditional KO (*Mpi*^CKO^) strains and pinpointed the cause of most phenotypes to be intestinal *Mpi* deficiency. As MPI-CDG is the best-studied human CDG treatable with mannose, we demonstrated mannose in drinking water rescues the phenotype in mice. The hypomorphic *Mpi^benadryl^* (*Mpi^ben^*) and Mpi-floxed (*Mpi^fl^*) alleles represent widely applicable tools to ablate and restore glycosylation in vivo and pave the way for discoveries of how glycobiology affects homeostasis and disease.

## Results

### Mapping the benadryl phenotype.

**Figure 1:**
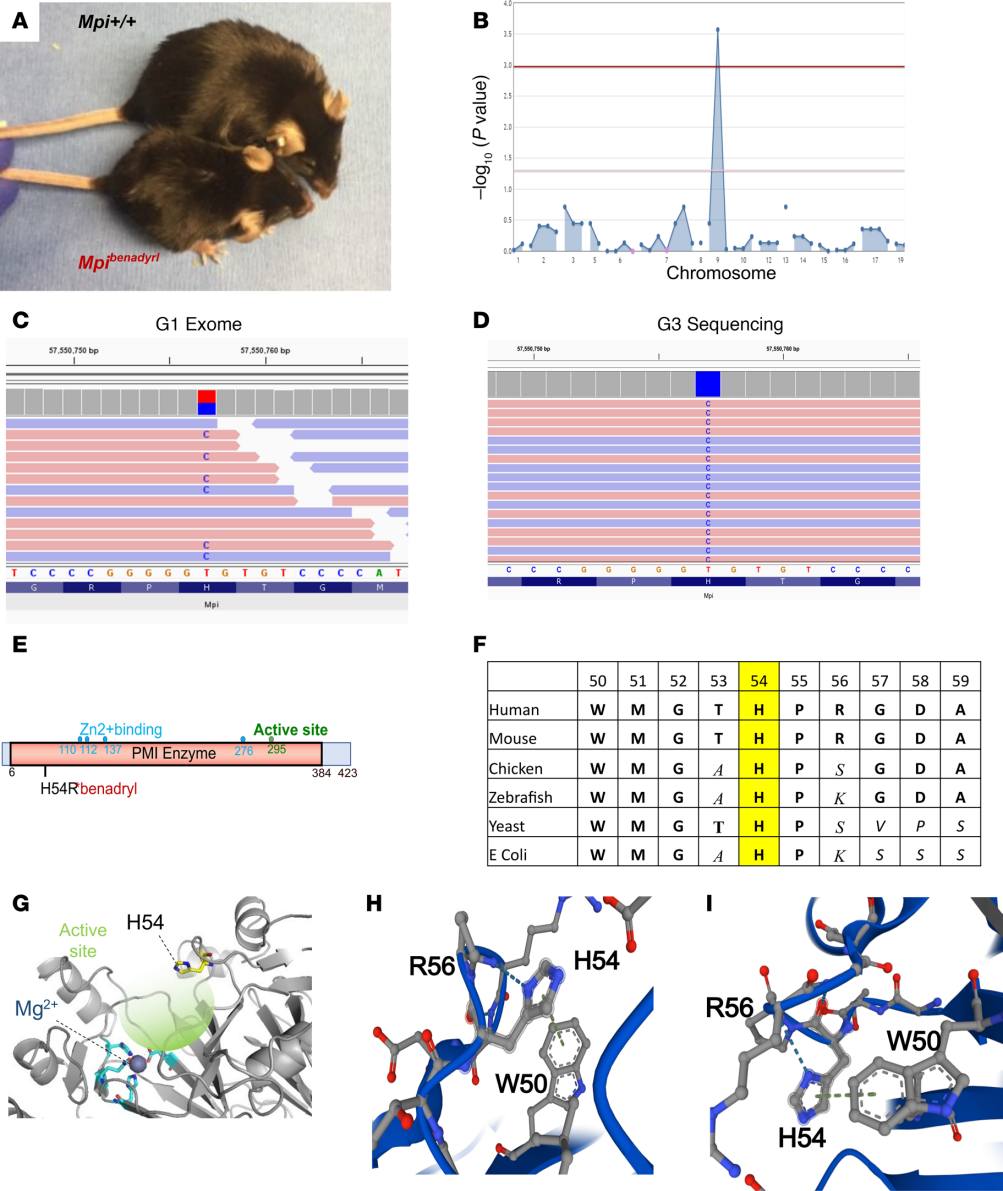
*Benadryl* has a viable, hypomorphic allele of *Mpi*, which causes a small body size and ruffled fur phenotype. (**A**) Photograph of *benadryl* mouse (bottom) compared with WT littermate. (**B**) Manhattan plot. Linkage of a “visible, abnormal phenotype” to a mutation in *Mpi* −log_10_ (*P* values) versus the chromosomal positions of mutations identified in the founder (generation 1, G1) of the affected pedigree, determined by (**C**) whole-exome sequencing and validated by (**D**) ion torrent next-generation sequencing of all G3 progeny. (**E**) Schematic of Mpi domains and the substitution of histidine to arginine at position 54 of 423 total amino acids. Numbers indicate amino acid positions. PMI enzyme, phosphomannose isomerase enzyme domain, also known as MPI. (**F**) Table showing conservation of amino acids at and adjacent to p.H54 (highlighted) across multiple organisms back to prokaryotes. (**G**) Structure of Mpi indicating H54 (yellow), active site binding pocket (highlighted in green; darker color is deeper portion and lighter color faces outward), and divalent cation-binding amino acids (blue). (**H** and **I**) Intermolecular N-N interaction of H54 with R56 and aromatic ring interactions of H54 and W50 shown from 2 orientations.

While performing forward genetic screening in third-generation (G3) mice from ENU-mutagenized founders, we detected several mice that had an allergic and visible phenotype (smaller size and ruffled or reduced fur) and named the strain *benadryl* (Figure 1, A and B). The immune phenotypes are being studied separately (JAS, UTSW, Dallas, Texas, USA, unpublished observations); this report will focus on the potential of a mouse model with N-glycosylation deficiency that is comparable to humans with MPI-CDG. Automated meiotic mapping linked the *benadryl* visible phenotype to a variant (p.H54R, Figure 1, C and D) in the gene *Mpi* (recessive inheritance pattern, *P* = 2.68 × 10^–4^, Figure 1, B and E). Manhattan plot analysis indicated no other chemically induced mutations linked to this phenotype (Figure 1B). Amino acids 50–55 are highly conserved as far back as prokaryotes (Figure 1F) and are located near the active site according to the crystal structure (Figure 1G) (23, 24). Arginine does not have the ring structure of histidine, but both are positively charged. Therefore, we investigated the intramolecular interactions of this site using AlphaFold modeling (24). One nitrogen of the His54 ring interacts with a terminal nitrogen in arginine, presumably through the positively charged arginine and unbound electron pair on the histidine nitrogen. While this arginine is not conserved as much as histidine is, the alternative amino acids are either positively charged (lysine) or polar (serine), which permit similar interactions. The tryptophan-histidine interaction aligns with an “edge-to-face” geometry, which is the most favorable noncovalent aromatic-aromatic interaction (Figure 1, H and I) (25). Thus, His54 may play an important role in maintaining MPI protein structure or substrate binding through interactions with neighboring conserved amino acids.

These factors led us to prioritize *benadryl* for further study by in vivo CRISPR validation. As KO of *Mpi* was previously described as embryonic lethal (8), we knocked in the *benadryl* allele (p.H54R) and bred the mice to have the homozygous mutation. These mice were confirmed to have the phenotype of ruffled, coarse fur and persistently lower BW (Figure 1B). Homozygotes were born at 60% of the expected Mendelian ratio (Supplemental Table 1; supplemental material available online with this article; https://doi.org/10.1172/jci.insight.180752DS1), and no compound heterozygotes were produced when the *benadryl* allele was crossed to a putative null frameshift allele (p.P55fs*15). Homozygous males were fertile, moderate breeders, but female homozygotes never became pregnant. The CRISPR knockin *benadryl* allele (*Mpi*^ben/ben^) was used for all subsequent experiments.

### Biochemical analysis.

**Figure 2:**
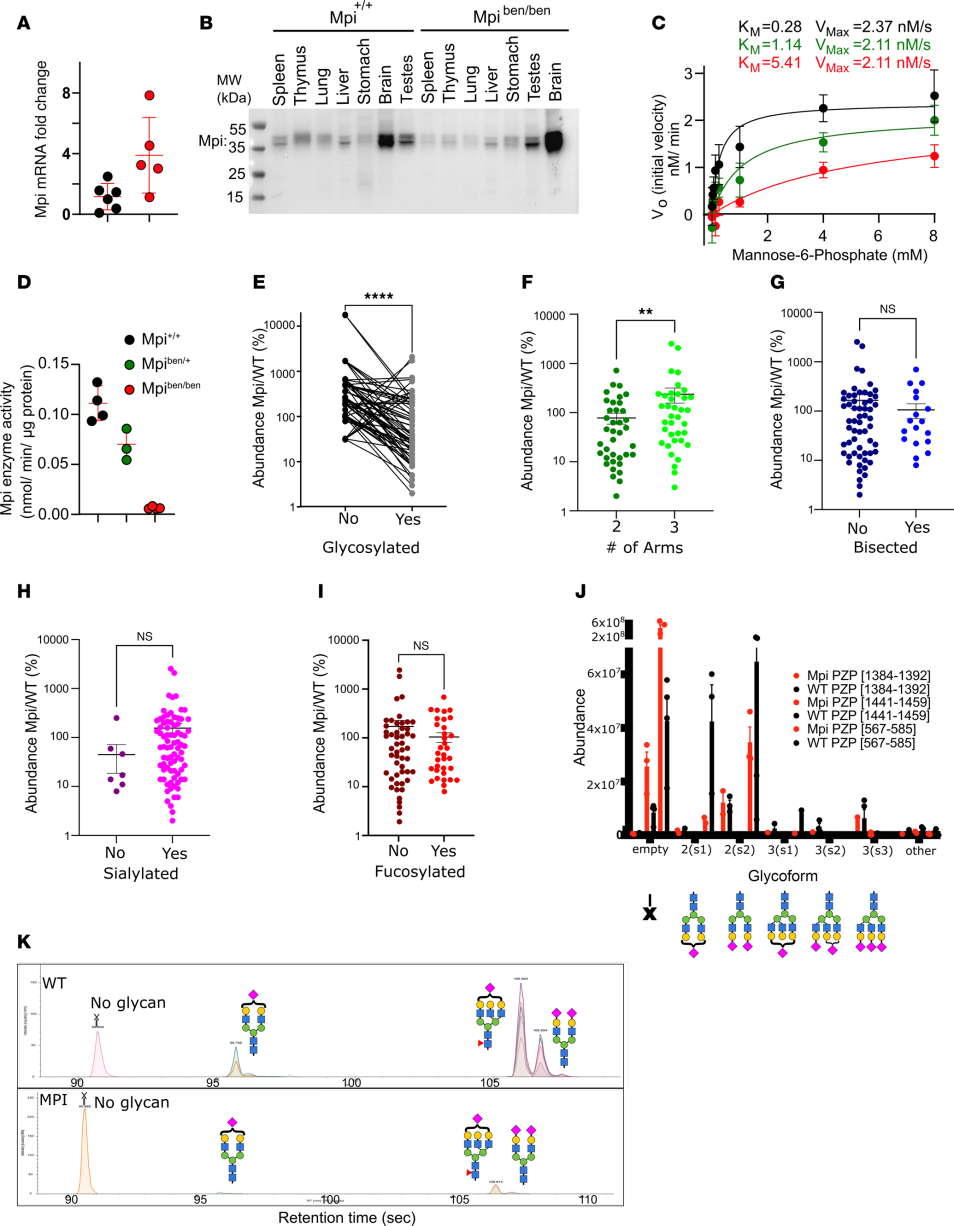
*Benadryl* variant decreases enzyme activity and substrate binding affinity, causing broad hypoglycosylation. (**A**) *Mpi* RNA analysis of peripheral mononuclear blood cells from *Mpi*^+/+^ or *Mpi^ben/ben^* mice; *Mpi* RNA normalized by levels of GAPDH. (**B**) Mpi protein expression in tissues from *Mpi*^+/+^ or *Mpi^ben/ben^* mice. (**C**) Enzyme activity was measured by generation of NADPH from mannose-6-phosphate (M6P) upon the addition of cell lysate obtained from peripheral blood. Initial velocity slope, V_0_ (nM/min), was measured across a range of M6P concentrations (0.306–8 mM). *P* values were determined by Student’s *t* test. Nonlinear regression of Michaelis-Menten kinetics was used by GraphPad to calculate V_Max_ and the *K_m_*. (**D**) Mpi enzyme activity was calculated as NADPH generated (nmol)/min/protein from cell lysate (μg) for *Mpi*^+/+^ (*n* = 4), *Mpi*^+/ben^ (*n* = 3), or *Mpi^ben/ben^* mice (*n* = 4). (**E**) Glycan mass spectrometry reveals there are more unoccupied glycan sites in *benadryl* mice than in WT mice; (**F**) there is a difference when comparing glycoforms with 2 arms compared with 3 or more arms, but it is not as appreciable; (**G**–**I**) there is no significant difference when comparing nonbisected to bisected forms, nonsialylated to sialylated forms, or non-fucosylated to fucosylated forms. (**J**) For example, an abundant glycoprotein, pregnancy zone protein (PZP), reveals more unoccupied sites in *benadryl* mice than in WT across multiple sites. (**K**) Mass spectrometry data for PZP site [567–585]. Data are representative of 2 (**A** and **B**) or 3 (**C** and **D**–**J**) experiments, and *n* = 3 *benadryl* and *n* = 4 WT mice (**E**–**K**). Black = WT, green = *Mpi*^+/ben^, and red = *Mpi^ben/ben^*. *P* values were determined by Student’s *t* test. **P < 0.01, ****P < 0.0001.

Quantitative PCR (qPCR) of splenocytes (Figure 2A) showed *Mpi* mRNA transcription was intact in *benadryl* tissue. We next investigated whether the mutation caused MPI protein destabilization and degradation, so we performed immunoblotting on Mpi in various mouse tissues (Figure 2B). Protein levels were similar across several tissues in WT and homozygous mice, including spleen, thymus, lung, liver, stomach, brain, and kidney (Figure 2B), using the best-performing Ab (MyBioSource, anti-MPI Ab, 4G9-B4-B8). Slightly lower Mpi expression in the spleen, thymus, and lung was not observed in an expanded series of replicates, so it was attributed to natural variation (Supplemental Figure 1). Since the MPI protein was transcribed and expressed at normal levels, we next examined whether reduced enzyme activity was responsible for the *benadryl* phenotype. Enzyme activity was determined by a modified assay that measures NADPH production with various concentrations of M6P substrate (8, 26). Michaelis-Menten first-order kinetics were observed and used to calculate the V_Max_ and *K_m_*. The V_Max_ was comparable for all zygosities, indicating similar total levels of Mpi protein, consistent with Western blot results. However, the *K_m_* was 20-fold higher in homozygotes than in WT littermates (Figure 2C). The increased *K_m_* indicates decreased substrate binding affinity. This 5% residual Mpi enzyme activity (Figure 2D) is comparable to the residual activity of affected humans with MPI-CDG who have 5%–12% enzyme activity (12, 20). Thus, the *benadryl* p.H54R allele impairs substrate binding to the active site (Figure 1G) and decreases enzyme activity.

We further tried to determine whether compound heterozygous *benadryl*/KO mice (estimated enzyme activity of 2.5%) were viable; however, none were produced among 51 pups from mating mice heterozygous for the KO and *benadryl* alleles (Supplemental Table 1). Therefore, the *benadryl* enzyme activity must reside within a narrow margin of low enzyme activity that produces a mouse with a phenotype but is still viable.

The expected functional impact of *Mpi* deficiency is decreased mannose precursors that would change glycan structure. Therefore, we performed glycan mass spectrometry. We sent samples to the Mayo Clinic Proteomics Core for analysis, but no transferrin signal was detected (possibly due to species-specific antibody). Thus, we performed untargeted glycoproteomics analysis. Glycopeptide analysis of littermate matched WT/heterozygous (Het) (*n* = 4) and *benadryl* (*n* = 3) mice yielded 288 unique glycopeptides, which were classified by branching, bisection, sialylation, fucosylation, and occupancy. Data showed *benadryl* mice had more unoccupied glycan sites than WT mice. There were 554% more unoccupied glycopeptide sites in *benadryl* mice than in WT mice (Figure 2E). Our findings are consistent with observations in humans where type 1 CDG transferrin glycan signatures lack N-glycans (27). Additionally, we evaluated N-glycan carbohydrate properties across 288 serum glycopeptides. When comparing WT with Mpi, we found an increased number of glycan arms (Figure 2F, *P* < 0.01) but no changes in bisecting N-Acetylglucosamine, sialylation, or fucosylation (Figure 2, G–I). Our untargeted serum glycoproteomics analysis did not detect transferrin glycopeptide sites. However, the glycopeptide sites in an abundant glycoprotein, pregnancy zone protein, alpha-2-macroglobulin like (PZP), had more unoccupied sites in *benadryl* mice than in WT mice (Figure 2J). Mass spectrometry data for PZP glycopeptide [567–585] demonstrated the >10-fold increase in unoccupied glycans at this site (Figure 2K).

### Glucose homeostasis.

**Figure 3:**
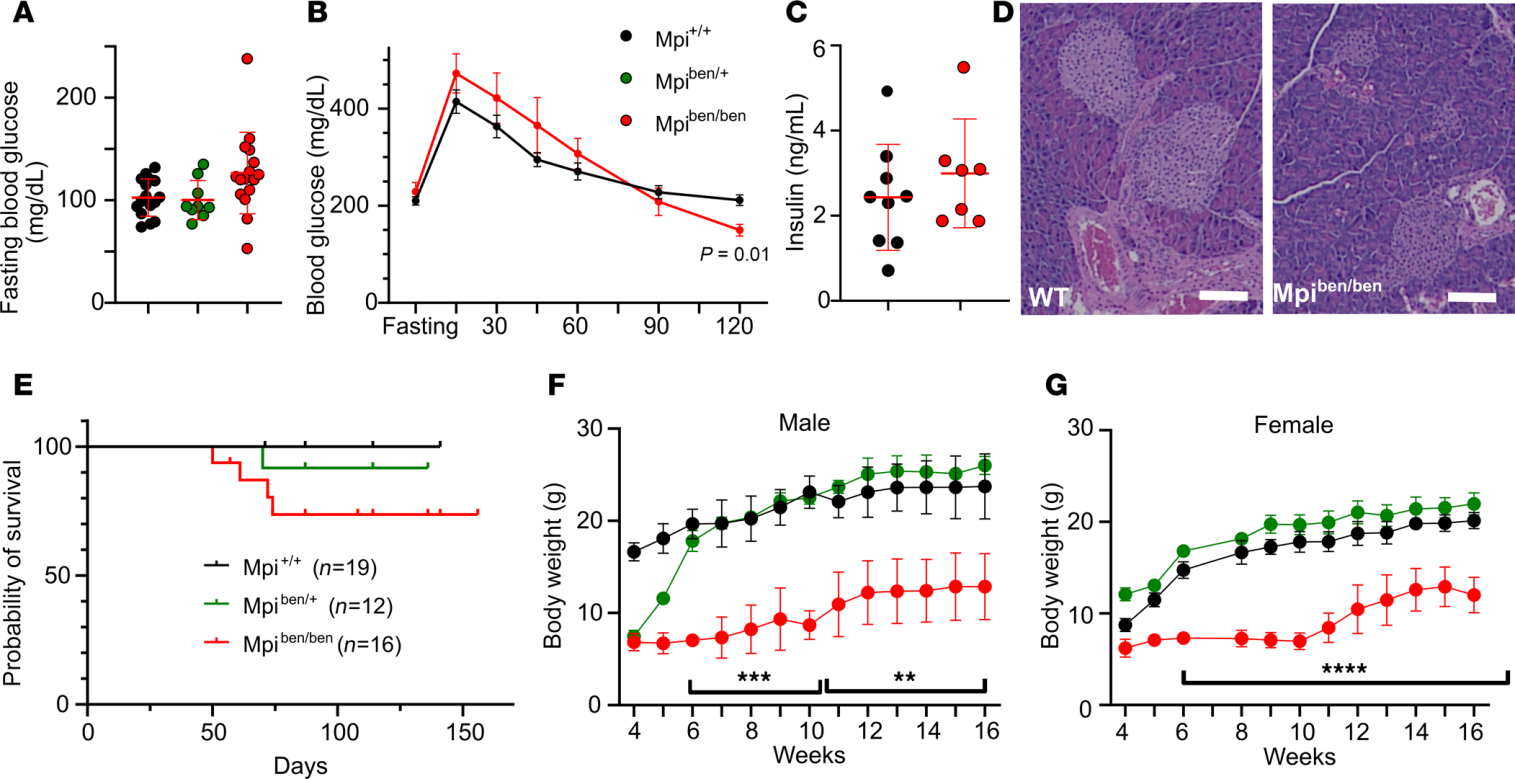
Metabolism is intact in *benadryl* mice. (**A**) Fasting blood glucose after 6 hours of fasting in *Mpi*^+/+^, *Mpi*^+/ben^, or *Mpi^ben/ben^* mice (*n* = 16, 9, 16). (**B**) Intraperitoneal glucose tolerance test was performed after 6 hours of fasting. Blood glucose was measured over 2 hours for *Mpi*^+/+^ and *Mpi^ben/ben^* mice (*n* = 11, 8). (**C**) Fasting insulin was measured by ELISA in *Mpi*^+/+^ and *Mpi^ben/ben^* mice (*n* = 9, 7). (**D**) H&E-stained histologic sections showing *Mpi*^+/+^ and *Mpi^ben/ben^* pancreatic islets. Scale bar is 50 mm. (**E**) Probability of survival (hash marks indicate mice lost to follow-up) and (**F** and **G**) BWs of male and female mice over time in *Mpi*^+/+^, *Mpi*^+/ben^, and *Mpi^ben/ben^* mice (male: *n* = 5, 2, 8, female: *n* = 7, 3, 5). Data combined from 2 experiments (**A** and **E**–**G**) or performed once (**B** and **C**). Data points represent individual mice. Error bars indicate SD. *P* values were determined by 1-way ANOVA with Tukey’s multiple comparisons test (**A**), 2-way ANOVA with Bonferroni’s correction (**B**), Student’s *t* test (**C**), and mixed effects analysis with Tukey’s multiple comparisons test (**F** and **G**). **P < 0.01, ***P < 0.001, ****P < 0.0001. Lines in histology images represent 100 µm.

Hyperinsulinemic hypoglycemia is a hallmark of MPI-CDG in humans (13, 28). However, this was not observed in *benadryl* mice. Metabolism was evaluated by measuring fasting blood glucose, which was equivalent among *benadryl* littermates in repeated experiments (Figure 3A). To further investigate glucose homeostasis, we performed an i.p. glucose tolerance test after fasting, but there were negligible differences in blood glucose between WT and *benadryl* littermates (Figure 3B). Furthermore, nonfasting insulin levels were normal in *benadryl* mice (Figure 3C), unlike in MPI-CDG. In addition, pancreatic islets were identified with normal histologic features with no apparent change in size or number (Figure 3D).

### Size and survival.

Just like humans with MPI-CDG, *benadryl* mice show decreased survival (13). Approximately 70% lived beyond 8 weeks (Figure 3E). However, we noticed survival could be increased if moist chow was provided; since many of the mice weighed less than 10 grams, they were too small to reach food pellets in the feeder. BW stayed low throughout the lifetimes of *benadryl* mice with a gradual increase with age (Figure 3, F and G). Their sparse hair phenotype was variable and improved or deteriorated with age with no obvious pattern. Histologic examination of the epidermis found an increase in sebaceous gland inclusions with no other major findings (Supplemental Figure 2A).

### Hematology.

**Figure 4:**
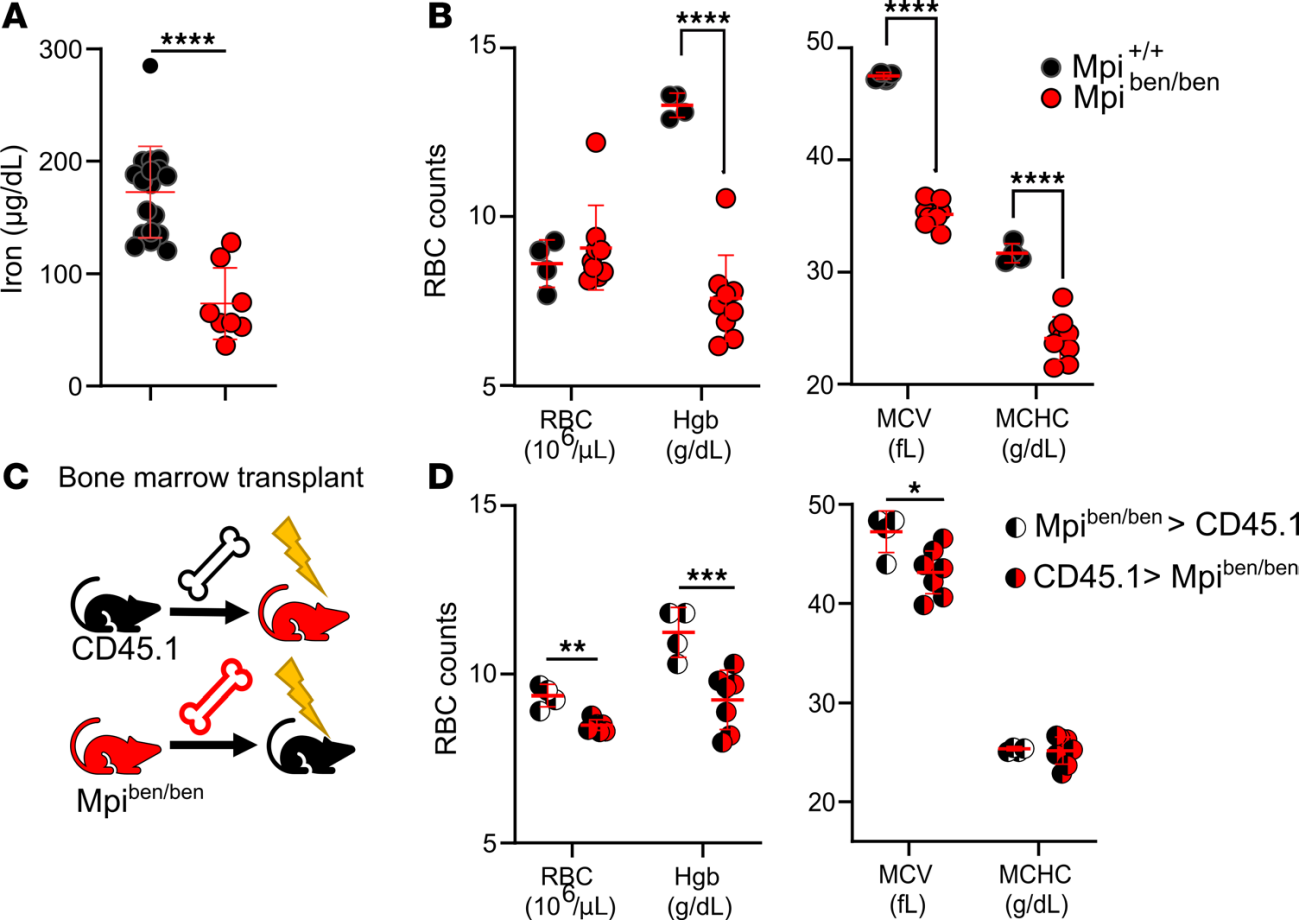
*Mpi* deficiency causes a microcytic, hypochromic iron deficiency anemia due to causes extrinsic to hematopoietic cells. (**A**) Serum iron was measured by the VITROS 350 System in *Mpi*^+/+^ and *Mpi^ben/ben^* mice (*n* = 18, 8). (**B**) RBC indices included RBC count, hemoglobin (Hgb), mean corpuscular volume (MCV), and mean corpuscular hemoglobin content (MCHC) in *Mpi*^+/+^ and *Mpi^ben/ben^* mice (*n* = 4, 9). (**C**) Reciprocal BM chimeras between CD45.1 (black) and *Mpi^ben/ben^* (red) mice (*n* = 4, 7) were performed by transplanting BM into lethally irradiated mice (lightning symbol). (**D**) RBC number, Hgb, MCV, and MCHC of CD45.1 mice transplanted with *Mpi^ben/ben^* BM (black and white circles: *n* = 4) or *Mpi^ben/ben^* mice transplanted with CD45.1 BM (red and black circle: *n* = 7). Data were combined from 2 experiments (**A**) or 1 experiment (**B**–**D**). Data points represent individual mice. Error bars indicate SD. *P* values were determined by Student’s *t* test (**A**, **B**, and **D**). *P < 0.05, **P < 0.01, ***P < 0.001, ****P < 0.0001.

Children with mutations in *MPI* experience anemia, so we investigated RBC phenotypes in *benadryl* mice. A complete blood count found that *benadryl* mice had microcytic (low mean corpuscular volume, MCV), hypochromic (low mean corpuscular hemoglobin [Hgb] concentration, MCHC) iron deficiency anemia (Figure 4, A and B). An increased RBC distribution width (RDW) is often attributed to compensatory hematopoiesis with an increase in the larger sized reticulocytes.

Reciprocal BM chimeras were created when *benadryl* BM was transplanted into lethally irradiated CD45.1 mice and CD45.1 BM was transplanted into *benadryl* homozygotes (Figure 4C). The Hgb and MCV were decreased in *Mpi^ben^* hosts with CD45.1 BM (Figure 4D). This shows *benadryl* mice are anemic even when they have WT blood cells present; therefore, the anemia phenotype is hematopoietic extrinsic (not caused by a blood cell defect). The iron deficiency anemia seen here suggests either bleeding or malabsorption as the source of anemia.

### Skin pathology.

*Benadryl* mice were noted to have variable hair loss, so skin pathology was examined. Aside from decreased frequency of hair follicles, the main feature noted was enlarged eosinophilic round inclusions in sebaceous cells (Supplemental Figure 2A). These may represent undigested unfolded proteins or lipid membranes. ENU mutagenesis has created multiple hair and skin defects in mice, in various patterns of hair loss. This variant resembles features of the *toku* mouse, which had lipid metabolism defects and loss of hair across the body but not the head (29, 30).

### Hepatic pathology.

**Figure 5:**
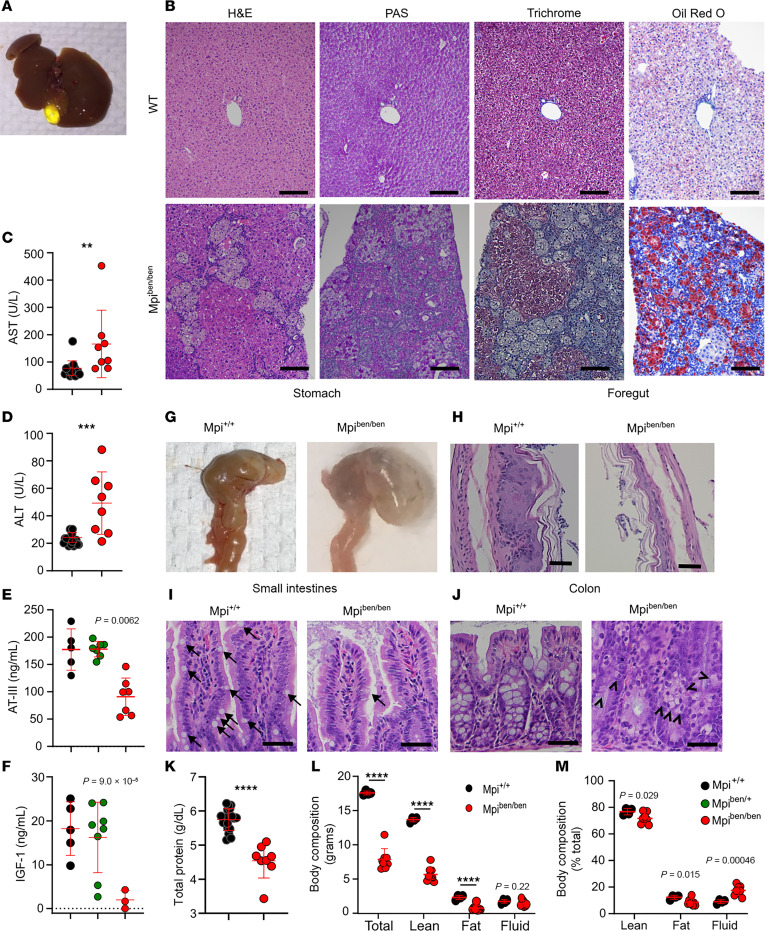
Liver and gastrointestinal tract are histologically and biochemically abnormal in *Mpi*-deficient mice. (**A**) Gross examination of *benadryl* mouse shows an enlarged gallbladder. (**B**) Representative histologic sections were stained with H&E, periodic acid–Schiff (PAS), trichrome, or Oil Red O in WT (top) or *Mpi^ben/ben^* mice (bottom). (**C** and **D**) Aspartate aminotransferase (AST) and alanine transaminase (ALT) were measured in *Mpi*^+/+^ and *Mpi^ben/ben^* mice (*n* = 18, 8 for AST/ALT). (**E**) Antithrombin III (ATIII) and (**F**) insulin like growth factor 1 (IGF-1) levels were measured in 10-month-old *Mpi*^+/+^, *Mpi*^+/ben^, and *Mpi^ben/ben^* littermates (ATIII: *n* = 5, 7, 7, IGF-1: *n* = 5, 8, 3). (**G** and **H**) Gross and microscopic images of *Mpi*^+/+^ and *Mpi^ben/ben^* stomachs, focusing on the foregut. (**I** and **J**) H&E-stained small and large intestine tissues from *Mpi*^+/+^ and *Mpi^ben/ben^* mice (arrows = goblet cells, arrowheads = immature goblet cells). (**K**) Serum total protein was measured by the VITROS 350 System in *Mpi*^+/+^ and *Mpi*^ben/ben^ mice (*n* = 18, 8). (**L** and **M**) Whole-body DEXA scan was used to measure lean soft tissue, adipose tissue, and fluid in total or as a percentage of total mass in *Mpi*^+/+^ and *Mpi^ben/ben^* mice (*n* = 4, 8). Data points represent individual mice from the aggregate of 2 experiments (**C**–**F** and **K**) or a single experiment (**L** and **M**). Black scale bars = 100 µm. Error bars indicate SD. *P* values were determined by Student’s *t* test (**C**, **D**, and **K**–**M**) or CRISPR-calculator (55, 63) for recessive inheritance. **P < 0.01, ***P < 0.001, ****P < 0.0001. Lines in histology images represent 100 µm.

A prominent feature of MPI-CDG is hepatic fibrosis, which causes elevated liver enzymes and decreased synthesis of certain coagulation factors (serpin family C member 1, also known as ATIII; protein C, inactivator of coagulation factors Va and VIIIa) and growth hormones (insulin like growth factor 1, IGF-1). Gross examination of the liver showed patchy pale areas and an enlarged gallbladder (Figure 5A). Histologically, these patchy areas along the edge of the liver demonstrated clusters of histiocytes filled with a substance that was negative to staining by both H&E and periodic acid–Schiff (PAS). The inclusion morphology was determined unlikely to be glycogen since it did not stain with PAS. Trichrome staining yielded blue staining, but a review by a pathologist showed this was due to enlarged macrophages rather than fibrosis. Oil Red O staining revealed a significant increase in lipids within the hepatocytes of *benadryl* mice compared with WT mice, particularly in periportal areas (Supplemental Figure 5C). This strongly suggests that MPI plays a role in regulating lipid metabolism in the liver and that when MPI expression is reduced, the ability of the liver to process lipids is compromised, leading to excessive accumulation of lipids within hepatocytes (Figure 5B). The extent of fibrosis was not prominent as in some case reports of MPI-CDG; however, those case reports often used histology from autopsy or transplant cases, reflecting the most severe stages of disease (14, 31). We verified biochemical consequences of hepatic pathology, including increased aspartate aminotransferase (AST) and alanine aminotransferase (ALT) and decreased ATIII and IGF-1 (Figure 5, C–F).

### Stomach and gallbladder pathology.

When dissecting *benadryl* mice, we observed the stomachs were enlarged with an antral cystic enlargement of the foregut (Figure 5G). Histologic examination revealed normal gastric epithelium with intact secretory cells, parietal cells, and glands. However, the outer layer of stratified squamous epithelium was thin in the foregut (Figure 5H). We hypothesized a smooth muscle (SM) defect may be present and investigated other tissues with substantial SM: the bladder and the gallbladder. No differences were noted in the bladder by histology (data not shown), but the gallbladders in *benadryl* mice were larger (Figure 5A) and demonstrated an attenuated, distended lining, like the stomach (Supplemental Figure 2). Weakened SM could lead to distensions in the stomach and gallbladder. Humans with MPI-CDG are reported to have cyclic vomiting, and while vomiting was not observed in the mice, it is possible that poor gastric SM contractility could explain the cyclic vomiting phenotype (13, 14). To determine whether the phenotypes observed were due to an SM defect, we created *Mpi*^fl^ mice with *loxP* sites flanking the fourth exon, leading to a frameshift variant when excised by Cre recombinase (Supplemental Figure 3, A–C). We created *Mpi*^fl/fl^ Myh11-ERT2^Cre^ (*Mpi*^ΔSM^) mice, which have a deletion of *Mpi* in SM cells upon tamoxifen injection. *Mpi*^ΔSM^ mice were observed for 3 months after injection, and no visible phenotype developed (JAS, UTSW, Dallas, Texas, USA, unpublished observations); we then collected tissues for histologic examination. Stomach, gallbladder, and bladder tissue were collected for histologic analysis, which showed no change in thickness of epithelial linings in *Mpi*^ΔSM^ compared to *Mpi*^fl/fl^ controls injected with tamoxifen (Supplemental Figure 2). Thus, another type of tissue defect must be responsible for the distended stomach and gallbladder phenotypes observed.

### Intestinal pathology.

Histologically, the small and large intestines were significant for having decreased goblet cells (Figure 5, I and J). Congenital diarrhea and PLE are prominent features of MPI-CDG, which are thought to cause hypoalbuminemia and edema (13, 14, 32). We verified *benadryl* mice exhibited 25% decreased serum protein levels (*P* < 0.0001, Figure 5K), a condition that, in patients, leads to water retention and systemic edema. Using dual-energy x-ray absorptiometry (DEXA) scans, we further verified the presence of 190% excess fluid (9.5% vs. 18%, *P* = 0.00046), consistent with edema in these mice. All the *benadryl* mice were smaller, likely due to lower lean soft tissue mass and adipose mass. When normalized as a percentage of the total BW, both adipose and lean soft tissue masses were marginally but significantly decreased in *benadryl* mice, but fluid body weight was doubled (*P* = 0.00046, Figure 5, L and M).

Much of the human disease is attributed to PLE, so we analyzed the intestines and fecal alpha-1-antitrypsin (A1AT). ELISA performed on A1AT from 2 manufacturers was uninformative and showed barely detectable levels in mice, possibly due to nonstandardized protocols. At the base of the *Mpi^ben^* colon, lighter staining cells with abundant pink cytoplasm were present. These may represent goblet cells that are dehydrated (Figure 5K). As MPI-CDG presents with congenital diarrhea and the intestinal histology was altered in *benadryl* mice, we aimed to evaluate the impact of Mpi deficiency, specifically in intestinal tissue. Therefore, we conditionally knocked out *Mpi* in intestinal epithelial cells using the *Villin*^Cre^ transgene (*Mpi*^fl/fl^ *Villin*^Cre^, *Mpi*^ΔVillin^).

To investigate the tissue-specific origin of the *benadryl* phenotypes, we produced the *Mpi*^fl^ strain with 2 *loxP* sites around exon 5. Combination with Cre recombinase would remove exon 5 (aa 163–223) and cause a frameshifted gene product starting after amino acid position 223 (Supplemental Figure 3, A–C). This frameshifted product would affect half of the enzyme structure, including the active site (Figure 1A), resulting in loss of function.

**Figure 6:**
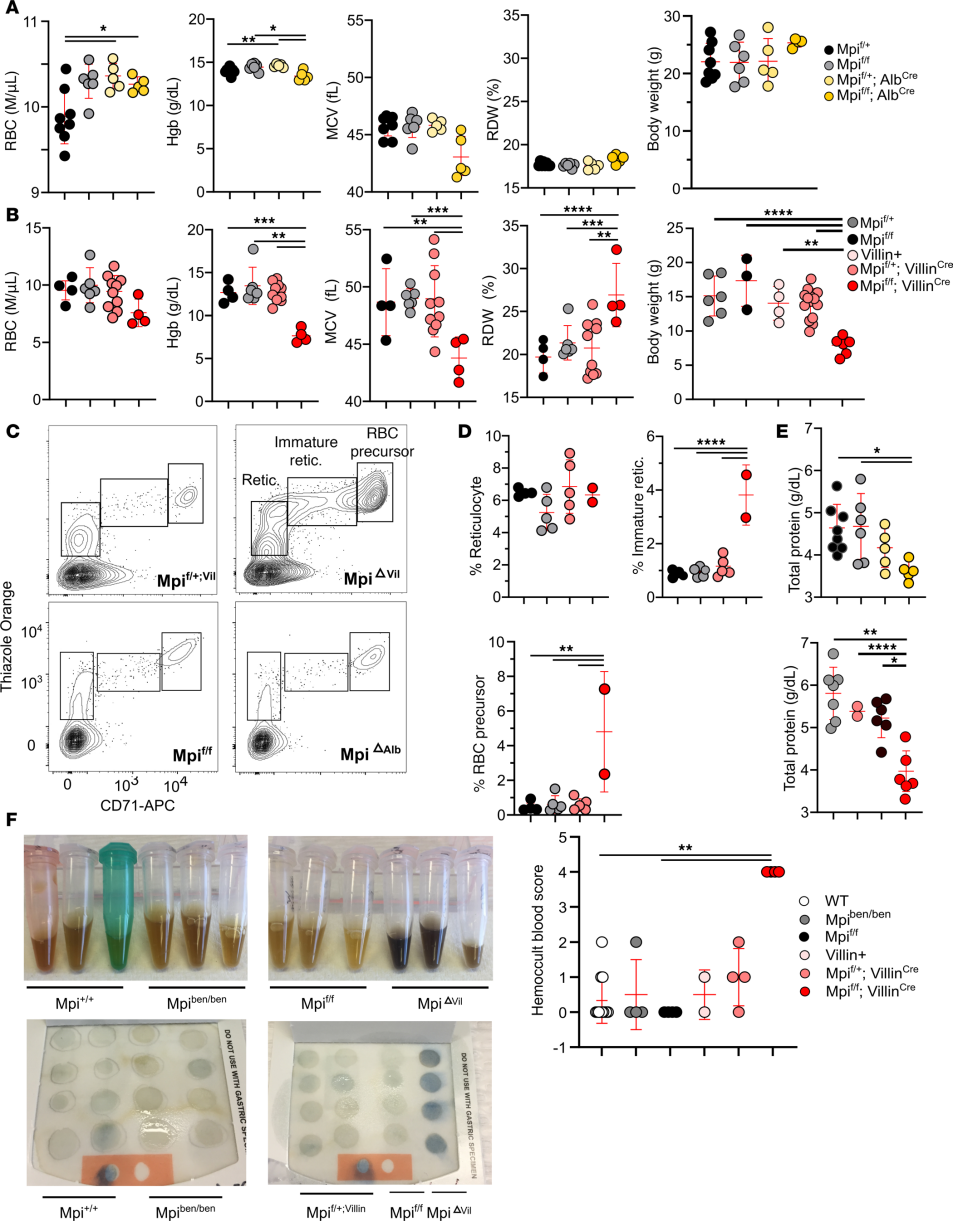
Anemia and low BW are caused by intestine-specific *Mpi* deficiency. (**A**) RBC indices measured are RBC count, Hgb, MCV, and MCHC in *Mpi*^fl/+^, *Mpi*^fl/fl^, *Mpi*^fl/+^ *Alb*^Cre^, and *Mpi*^fl/fl^ *Alb*^Cre^ mice (*n* = 8, 6, 5, 5), along with BW (*n* = 8, 6, 5, 4). (**B**) RBC count, Hgb, MCV, and MCHC in *Mpi*^fl/+^, *Mpi*^fl/fl^, *Mpi*^fl/+^ *Villin*^Cre^, and *Mpi*^fl/fl^ *Villin*^Cre^ mice (*n* = 4, 6, 10, 4) and BW (*n* = 6, 3, 4, 15, 6). (**C**) Representative flow cytometry plots using thiazole orange and CD71 to determine reticulocyte count in peripheral blood and (**D**) quantification of RBC precursors and reticulocytes. (**E**) Serum total protein in *Mpi*^fl/+^, *Mpi*^fl/fl^, *Mpi*^fl/+^ *Alb*^Cre^, and *Mpi*^fl/fl^ *Alb*^Cre^ mice (*n* = 8, 6, 5, 5) and *Mpi*^fl/fl^, *Mpi*^fl/+^, *Mpi*^fl/+^ *Villin*^Cre^, and *Mpi*^fl/fl^ *Villin*^Cre^ mice (*n* = 7, 2, 6, 6). (**F**) Stool lysates for *Mpi*^+/+^, *Mpi^ben/ben^*, *Mpi*^fl/fl^, and *Mpi*^ΔVillin^ mice with corresponding Hemoccult card reactions below. Hemoccult test results quantified on the right, WT, *Mpi^ben/ben^*, *Mpi*^fl/fl^, *Villin*^Cre^, *Mpi*^fl/+^ *Villin*^Cre^, and *Mpi*^fl/fl^ *Villin*^Cre^ mice (*n* = 12, 4, 4, 2, 4, 4). Data points represent individual mice from the aggregate of 2 experiments (**A**, **B**, **E**, and **F**) or 3 independent experiments (**C** and **D**). Error bars indicate SD. *P* values were determined by 1-way ANOVA with post hoc Tukey’s test (**A**–**F**). *P < 0.05, **P < 0.01, ***P < 0.001, ****P < 0.0001.

Notably, *Mpi*^ΔVillin^ mice reproduced many of the phenotypes of *benadryl* mice, including small, frail appearance and microcytic, hypochromic anemia. Since patients with MPI-CDG often exhibit liver fibrosis and transaminitis and *benadryl* mouse liver histology was altered, we aimed to evaluate the impact of Mpi deficiency, specifically in liver tissue. Therefore, we conditionally knocked out Mpi in intestinal epithelial cells using Alb^Cre^. The liver-specific CKO mouse model, *Mpi*^ΔAlb^, had largely normal RBC parameters (Figure 6A; RBC *P* < 0.05 *Mpi*^fl/fl^ vs. *Mpi*^ΔAlb^; Hgb *P* < 0.05 *Mpi*^fl/fl^ vs. *Mpi*^ΔAlb^), while *Mpi*^ΔVillin^ mice had worse anemia (Figure 6B, *P* < 0.01–0.0001). Flow cytometry to detect the immature RBCs (CD71^+^, thiazole orange^+^) showed a significant increase in immature reticulocytes in *Mpi*^ΔVillin^ mice compared with less than 2% in controls (Figure 6, C and D, *P* < 0.0001). BW and overall appearance were also normal in *Mpi*^ΔAlb^ mice, but body weight was decreased in *Mpi*^ΔVillin^ (8.8 g, 57% reduced vs. *Mpi*^fl/+^ *Villin*^Cre+^, *P* < 0.001). Serum protein levels were decreased by 22%–24% for both *Mpi*^ΔAlb^ and *Mpi*^ΔVillin^ mice compared with their *Mpi*^fl/fl^ littermates (Figure 6E, *P* < 0.05). With several phenotypes arising from defective intestinal Mpi, we suspected PLE would be detected. However, fecal A1AT levels were not significantly different in *Mpi*^ΔVillin^ mice (Supplemental Figure 4). Thus, we attempted to measure intestinal permeability by performing oral gavage with FITC-dextran. However, in the *Mpi^ben/ben^* mice tested, there was no significant difference in serum FITC-dextran levels detected compared with WT controls (data not shown). This assay has limitations as it often involves significant damage (such as during dextran sulfate sodium–induced colitis) to allow significant amounts of FITC-dextran to cross the intestinal lining. The *Mpi*^ΔVillin^ mice were too small to perform the FITC-dextran assay safely.

The fecal samples from *Mpi*^ΔVillin^ had a dark color reminiscent of chemically lysed blood, so we hypothesized blood may be present. When fecal samples from WT, *Mpi^ben^*, *Mpi*^fl/fl^, and *Mpi*^ΔVillin^ mice were tested on Hemoccult blood cards, the *Mp*i^ΔVillin^ samples were strongly reactive (score of 4 vs. 0–2 for controls) for blood in the stool (Figure 6F). This blood loss probably accounts for the pronounced iron deficiency anemia in *Mpi*^ΔVillin^ and *benadryl* mice. These results suggest intestinal blood loss is the reason for blood loss, but the data are not entirely definite.

The human disease MPI-CDG has characteristic histologic abnormalities in the colon, including large, dilated crypts and expanded lymphatic ducts (lymphangiectasia) (32). While the dilated crypts may resemble crypt abscesses, such as those seen in inflammatory bowel disease, only sloughed-off epithelial cells, and not inflammatory cells such as neutrophils, are present. Dilated crypts were not seen in *benadryl* colons, but *Mpi*^ΔVillin^ colons had multiple prominent crypt dilations. Crypt dilations were circular to oval in shape and 50–100 μm in size. They were found both alone and in clusters along the length of the colon with no predilection for the proximal versus distal colon.

### Tissue-specific pathology in Mpi^CKO^ mice.

**Figure 7:**
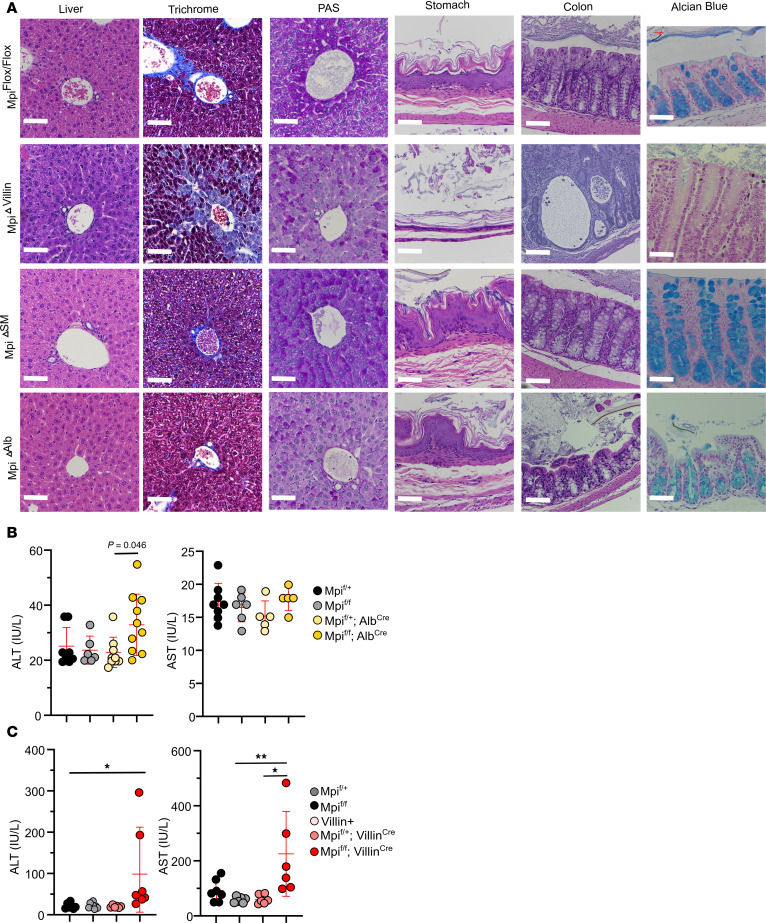
Histopathology of stomach and colon linked to intestinal *Mpi* deficiency. (**A**) Histological sections of the liver visualized with H&E stain (left), trichrome stain (middle), and PAS stain (right); stomach and colon were stained with H&E, and the colon was further stained with Alcian blue to highlight mucins. Scale bar is 50 mm. (**B**) Serum ALT and AST were measured in *Mpi*^fl/+^, *Mpi*^fl/fl^, *Mpi*^fl/+^ *Alb*^Cre^, and *Mpi*^fl/fl^ *Alb*^Cre^ mice (*n* = 8, 6, 5, 5) and (**C**) *Mpi*^fl/+^, *Mpi*^fl/fl^, *Mpi*^fl/+^ *Villin*^Cre^, and *Mpi*^fl/fl^ *Villin*^Cre^ mice (*n* = 7, 6, 6, 6). Histologic images are representative of 5 independent mice (**A**). Data points represent individual mice from the aggregate of 3 experiments (**B** and **C**). Error bars indicate SD. *P* values were determined by 1-way ANOVA with post hoc Tukey’s test. *P < 0.05, **P < 0.01. Lines in histology images represent 100 µm.

Despite MPI being highly expressed in the liver, Mpi liver-specific loss does not cause significant disease in mice. Contrary to our hypothesis, the *Mpi*^ΔAlb^ mice did not replicate the hepatic phenotypes of *benadryl* mice or patients with MPI-CDG, where liver fibrosis is a prominent feature (Figure 7, A and B). As hepatic disease and biliary distension were not reproduced in *Mpi*^ΔAlb^ and *Mpi*^ΔSM^ mice, yet *Mpi*^ΔVillin^ mice reproduced several other features of MPI-CDG, we examined the mice for any phenotypes unaccounted for by the CKOs. In the *Mpi*^ΔVillin^ strain, liver enzyme levels were markedly elevated, with an AST range of 78–458 for *benadryl* and with most WT values 55–92 except for 1 outlier (Figure 7C, AST *P* = 0.0016). ALT levels were on average twice as high in *benadryl* mice (50 vs. 24.5 IU/mL, *P* = 0.0001). Hepatic histology was next examined, since cirrhosis, tortuous distortion of the bile ducts, and enlarged portal tracts have been found in humans with MPI-CDG (13, 33). This morphology occurs as a developmental defect of ductal plate malformation and remodeling during bile duct formation. While this phenomenon was not observed in the *benadryl* strain, *Mpi*^ΔVillin^ livers exhibited altered coloration on trichrome stain and portal tract malformation (Figure 7A). These findings suggest *Mpi*^ΔVillin^ models hepatic defects of MPI-CDG even better than *benadryl* mice.

Because the stomach and gallbladder were unchanged in the SM-CKO strain *Mpi*^ΔSM^, we systematically examined histology in other CKOs: *Mpi*^ΔAlb^ and *Mpi*^ΔVillin^ (Figure 7A). The *Mpi*^ΔAlb^ mice did not have any gallbladder distension or bile duct pathology, but the *Mpi*^ΔVillin^ mice had enlarged gallbladders and stomachs with a thin lining (Figure 7A and Supplemental Figure 2). These defects were visible upon dissection and clearly observed microscopically along with abnormal bile ducts in *Mpi*^ΔVillin^ livers. The squamous cell foregut lining of the *Mpi*^ΔVillin^ stomach was attenuated with only a few cell layers of thickness. Likewise, the *Mpi*^ΔVillin^ gallbladder epithelial layer was only 1 cell layer thick (Supplemental Figure 2). Notably, villin 1 (VIL1), a brush border cytoskeleton protein, is expressed in the epithelial linings of the stomach and gallbladder. Therefore, the selective deletion of *Mpi* in VIL1-expressing tissues causes attenuated epithelial linings in the stomach and gallbladder. These data support the hypothesis that many of the phenotypes of *benadryl* mice arise from gastrointestinal pathology.

Loss of goblet cells has not been described previously in humans, but examination of primary literature images confirms reduction in goblet cells. Goblet cells produce large levels of mucus glycoproteins, which are heavily glycosylated. Alcian blue stains positive for goblet cells and verified reduction in goblet cells in *benadryl* small and large intestines (Figure 7A). Furthermore, goblet cells were almost entirely absent in *Mpi*^ΔVillin^ intestines. Mucus provides a barrier from commensal bacteria, and mucus loss is associated with inflammatory bowel disease in mice, so this higher level of intestinal pathology in *Mpi*^ΔVillin^ is likely linked to these mice having fewer goblet cells compared with *Mpi^ben^* homozygotes. In addition to lacking mucin-producing goblet cells, the colon had crypt dilations, as with human MPI-CDG. These villi had expanded interstitial space, perhaps due to edema or lymphatic vessel blockage.

### Mannose treatment reverses effects of Mpi deficiency.

**Figure 8:**
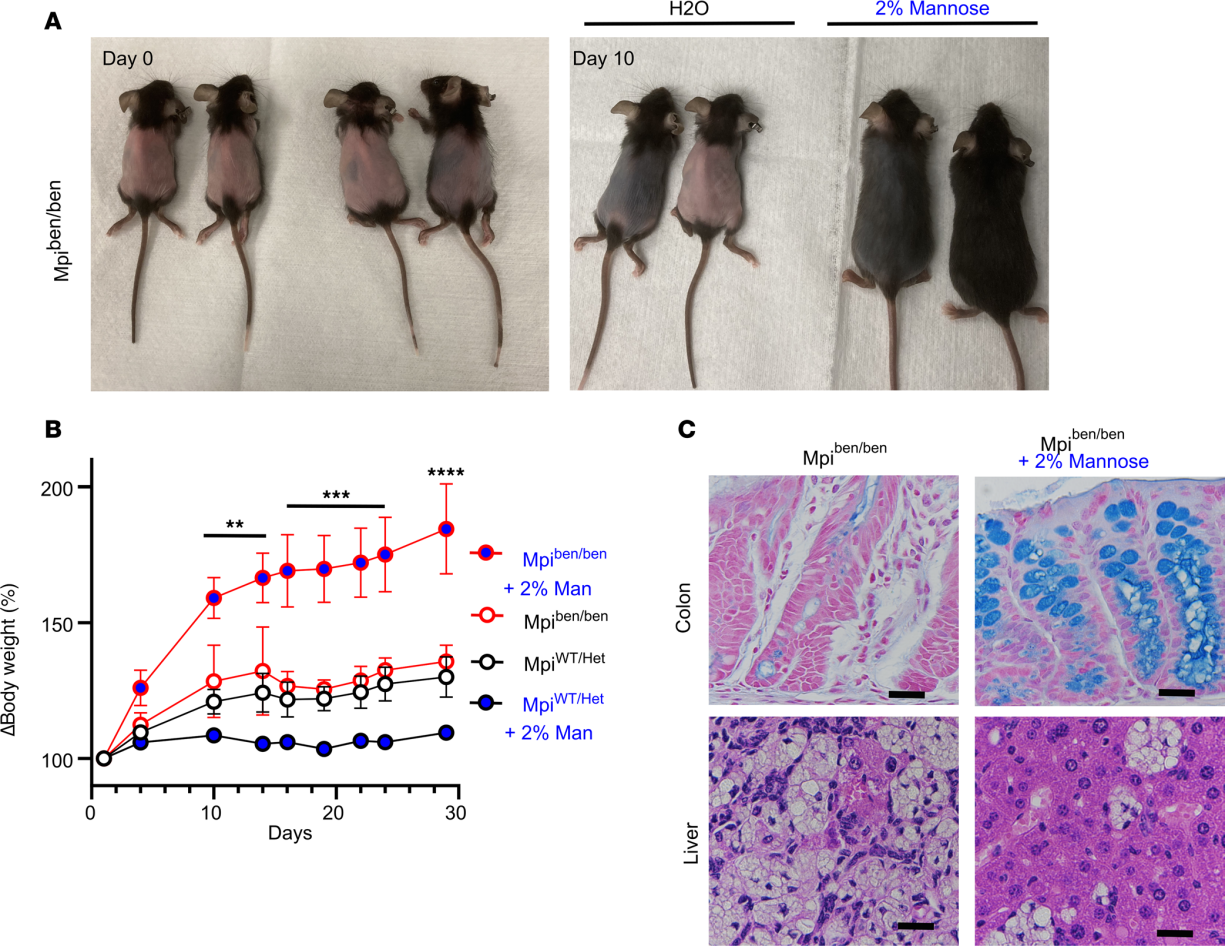
Oral mannose therapy rescues *Mpi* deficiency. (**A**) *Mpi*^ben/ben^ mice were treated with 2% mannose in drinking water for 30 days total. Photographs were taken on day 0 (left) and day 10 (right). (**B**) Relative changes in body weight of *Mpi*^WT/Het^ (*Mpi*^+/+^ or *Mpi*^ben/+^) or *Mpi*^ben/ben^ mice after treatment with water (white fill, *n* = 5, 4) or 2% mannose (blue fill, *n* = 2, 6). (**C**) Microscopic images of colon and liver tissue from *benadryl* mice treated with water (left) or 2% mannose (right) for 30 days (200× original magnification, representative of 4–5 mice). Points represent mean values for mice that are representative of 3 experiments (**B**). Error bars indicate SEM. **P < 0.01, ***P < 0.001, ****P < 0.0001. Lines in histology images represent 25 μm.

*Mpi* deficiency (MPI-CDG) is one of the only glycosylation precursor defects treatable with carbohydrate supplementation (15, 28, 34). Normally, mannose is present at levels 100-fold lower than glucose,and 5–10 times lower in *MPI*-deficient patients (35, 36). Exogenous mannose can increase serum levels of mannose 2- to 5-fold (15, 37) and be imported into cells and converted into M6P by hexokinase to bypass a malfunctioning MPI enzyme (37). We tested whether this feature of MPI-CDG was also present in *benadryl* mice by giving 2% mannose in drinking water for 1 month. Hair rapidly normalized within 10 days, and BW significantly increased within 10 days (Figure 8, A and B, *P* < 0.01). BW continued to improve until it reached levels consistent with WT and heterozygous littermates at 30 days (*P* < 0.0001). Histologic examination of the liver after 30 days of mannose treatment showed a reduction in the size and number of abnormal macrophage aggregates (Figure 8C). Likewise, mannose ameliorated intestinal pathology by restoring goblet cells to normal levels, as seen by Alcian blue staining (Figure 8C).

## Discussion

We describe a viable mouse model that recapitulates most features of the rare congenital glycosylation disorder, MPI-CDG, and has potential for further insight into glycan biology. The significance of glycosylation in physiologic processes has been underappreciated, partially due to the difficulty in studying these diverse modifications on proteins (38). Challenges have included the complexity of computational mass spectrometry analysis and the high diversity in glycosylation arrangements. Modeling CDG in vivo has proven challenging; almost all CDG KO models are embryonic lethal, likely due to the global impact of glycosylation on many physiologic processes and neurologic development (38–40). Similarly, no humans with 0% MPI enzyme activity have been found. Thus, many glycosylation studies have been performed in vitro (41).

In this study, we introduced a point mutation in *Mpi* found from a forward genetic screen (p.H54R) in mice. This point mutation led to an immune phenotype, *benadryl*, which is similar to MPI-CDG. The immune phenotype of *benadryl* mice is related to the absence of allergic diseases or responses; thus, the mouse model was named *benadryl*. Associated immune phenotypes do not cause immunodeficiency, as has been seen in some CDGs with recurrent infections. We use CRISPR knockin mice and CKO mouse models and perform rescue experiments with mannose to reach this conclusion. Validation with data from human patients with MPI-CDG supports the use of *benadryl* mice to model the human condition, MPI-CDG.

The mice with this mutation are still viable because of approximately 5% residual MPI enzyme activity and decreased substrate binding (increased *K_m_*). From the MPI crystal structure, we concluded the substrate is likely blocked from the catalytic site by the substituted arginine (p.H54R) (23). The p.H54R variant produced homozygous mice at a rate of 60% of the expected Mendelian ratio. We also created an *Mpi*^fl^ allele for CKO. We found that just as in humans with MPI-CDG, *benadryl* mice had a phenotype that could be rescued with oral mannose. While glycosylation affects most physiologic systems, we found in this study that downregulation of MPI leads to phenotypes that are attributable to intestinal epithelial hypo–N-glycosylation. One of the principal symptoms in human patients is diarrhea; here, we found loss of goblet cells in the large intestines of *benadryl* mice, which could lead to intestinal malabsorption or PLE (13, 35). The intestinal phenotype was validated by using a *Villin*^Cre^ CKO that had not only goblet cell loss but also the characteristic villi dilations and cysts seen in patients with MPI-CDG (32). While *benadryl* mice did not have intestinal bleeding, *Mpi*^ΔVillin^ mice consistently had the highest levels of fecal blood loss. However, this did not translate into elevated fecal A1AT levels in the stool, which is used to determine whether PLE is present. Since there is not a standardized method for this type of assay in mice, it is possible the phenotype was missed because of sample processing defects. Despite lacking PLE, *benadryl* mice displayed low total protein and minimal edema by DEXA scan.

The *benadryl* and *Mpi*^fl^ mouse models provide opportunities to discover insights into the fundamentals of glycobiology in vivo. Experiments performed using *Mpi*-deficient strains could have an important impact on fundamental glycobiology, physiology regulated by glycans, and preclinical therapeutic modeling for patients with rare disorders of glycosylation.

Some of the most significant phenotypes in *benadryl* mice were defects in RBCs. We found a microcytic, hypochromic anemia consistent with iron deficiency anemia. However, an increased RDW indicated increased reticulocytes that were larger from BM that was compensating by producing more RBC precursors. Flow cytometry verified that an abnormally large number of RBC precursors (CD71^hi^, thiazol orange^+^) were present in the peripheral blood (42). Anemia is common in patients with MPI-CDG and may be due to defective iron absorption or blood loss secondary to intestinal pathology (13). We further performed a reciprocal BM between *benadryl* and WT mice to examine whether the RBC phenotype was due to a process intrinsic or extrinsic to hematopoietic cells (43, 44). The anemia phenotype was improved when *benadryl* mice received WT BM; however, the strength of the phenotype can be difficult to evaluate in mice that have been irradiated. Hematologic evaluation of *Mpi*^ΔVillin^ mice demonstrated profound microcytic, hypochromic anemia with an increased RDW; flow cytometry verified reticulocytosis. Therefore, we conclude that anemia in *benadryl* mice may be multifactorial. The primary cause is likely due to factors extrinsic to hematopoietic cells, principally intestinal *Mpi* deficiency.

Hepatic fibrosis, transaminitis, and consequently decreased synthesis of coagulation factors are other prominent features of MPI-CDG (14, 31, 32). While liver enzymes were elevated by 3 times in *benadryl* mice compared with WT mice, the *Mpi*^ΔAlb^ mice did not have significantly higher liver enzyme levels compared with littermate controls. Elevated liver enzyme levels in *Mpi*^ΔVillin^ mice could be explained by hepatitis induced by intestinal transmigrating bacteria (45–47). Altered intestinal barrier and microbiome will be reported separately. This link could explain why the intestine-specific CKO causes transaminitis.

Histologic analysis did not show hepatic fibrosis, but it did show an increase in periportal enlarged macrophages, which were filled with lipids. Mannose treatment shrank the size of these hepatic macrophage accumulations. This improvement from mannose therapy differs in human MPI-CDG, where liver disease is not affected by mannose therapy (20, 35) and requires liver transplantation (13). While we could not reproduce the fibrosis phenotype, we verified other findings in MPI-CDG, such as decreased ATIII and IGF-1 (Supplemental Figure 5, A–C), which are linked to hepatic dysfunction.

Gross examination of *benadryl* stomachs revealed a transparent, cystic enlargement of the anterior portion of the stomach. Histologic examination of the stomachs was significant for thinning of the squamous epithelium in the foregut. Normally, several layers of squamous epithelium provide structural integrity in the murine foregut, but the lining is stretched and atrophied in *benadryl* mice. Although we suspected this could arise from an SM defect, the *Mpi*^ΔSM^ did not have any signs of an atrophic stomach or foregut by gross or histologic examination. However, *Mpi*^ΔVillin^ mice had the same thinned lining of the foregut as *benadryl* mice. Although VIL1 is expressed in the gland cells of the stomach, it is unclear how the squamous epithelial layer was affected. As there is no VIL1 expression in the foregut, the thinned lining may be due to decreased bowel movement, which leads to backup of food in the stomach. Alternatively, some nutrients essential for gastric integrity are poorly absorbed, causing the defect. While humans do not have squamous epithelium or a foregut, cyclic vomiting is a common symptom of MPI-CDG; (48) this could be explained by decreased bowel motility leading to delayed gastric emptying and increased risk for vomiting (49, 50). Another explanation could arise from a defect in gastric epithelial cells, where VIL1 is expressed, which would lead to a mucus defect that could manifest as increased acidity and ulceration, as has been found in 2 patients with *MPI* deficiency (33, 51).

Similarly, we noticed gallbladders of *benadryl* and *Mpi*^ΔVillin^ mice were enlarged on gross examination. This was also hypothesized to be due to an SM defect; however, *Mpi*^ΔSM^ gallbladders had normal size, shape, and histologic features after transgene activation with tamoxifen. Furthermore, the bladder, another organ that would be affected by SM defects, was not enlarged under gross examination and did not have thin or atrophic walls by microscopic examination. Since the gallbladders in *Mpi*^ΔVillin^ mice were enlarged and atrophic, we suggest there may be some extrinsic mechanism that prevents the gallbladder from emptying. As VIL1 is expressed in the gallbladder epithelium, it is possible that *Mpi* deficiency causes an intrinsic cell defect.

Liver damage was limited to bile duct malformation and only occurred in *Mpi*^ΔVillin^ mice with distended gallbladders. Therefore, we suspect a link between intrahepatic bile duct malformation and the extrahepatic biliary system (gallbladder), which share similar molecular mechanisms of development (52). VIL1 is highly expressed in the gallbladder epithelium, which would explain the phenotype in the *Mpi*^ΔVillin^ strain (53). The distended gallbladders may be unable to contract and release bile, which could then back up into the liver and put pressure on bile ducts, leading to the malformations observed.

Although one of the most prominent features of human *MPI* deficiency is hyperinsulinemic hypoglycemia, metabolic functions of *benadryl* mice were unaffected. Fasting blood glucose and glucose challenge tests were normal. Serum insulin levels and pancreatic islet size and morphology did not reveal any differences. Insulin responses can vary by mouse strain, so using a non-C57BL/6 mouse may produce different results; though this is not a part of our future directions, it would be interesting for other groups to explore in the future (54). This is an example of how mouse models are not always perfect models to identically recapitulate human diseases. For example, in atherosclerosis, the primary lesion sites differ between mice and humans (aorta vs. coronary arteries) (55). Also, in cystic fibrosis, the mice primarily develop pancreatic instead of lung dysfunction (56).

Some differences in the mouse and human phenotype raise further questions about the human CDG that we intend to address with the *benadryl* model in future investigations. For instance, the rate at which mice recovered while on mannose supplementation was 4 to 6 times faster than the typical clinical response time of 1 to 2 months for *MPI*-deficient patients taking supplemental mannose. Several factors differ in the laboratory mice that may influence their response to therapy compared with humans. For example, fructose-1-phosphate inhibits Mpi; fructose is essentially absent in mouse chow diets but can be as high as 20% of a Western diet (57). Excess fructose can drive mannose production through residual Mpi activity. Another conundrum that has yet to be elucidated is why neurologic function is intact in *MPI* deficiency but is affected in other downstream CDGs. We confirmed that expression of MPI is higher in the brain (58), so residual function of higher MPI protein levels likely produces sufficient glycosylation in the tissue. Additionally, the loss of goblet cells links N-glycosylation to mucus production and intestinal protection. It is also unclear what the mechanistic role of N-glycosylation in vivo is, since the major mucin glycoprotein, Muc2, is heavily O-glycosylated (52, 54, 55). We are addressing these questions in our future investigations using this mouse model of Mpi deficiency to impact therapy in affected patients.

An in vivo tool such as these mouse strains that lead to generalized hypoglycosylation is a useful starting point to determine the role of glycosylation in specific proteins. Just as knocking out a gene demonstrates an essential function, removing glycosylation would do the same for a protein. However, it is tedious to target each glycan site of a protein. The *benadryl* strain blocks mannose synthesis at an early stage, globally decreasing N-linked glycosylation. Thus, *benadryl* mice are a useful model for examining in vivo effects of a range of glycosylated proteins.

## Methods

### Sex as a biological variable.

We included equal numbers of male and female mice littermates. We initially evaluated whether sex contributed as a biological variable (Figure 3, F and G), but the strong Mendelian nature of the mutation had the largest effect. Data from both sexes were aggregated for all subsequent experiments.

### Mice.

Our study examined male and female animals, and similar findings are reported for both sexes. A C57BL/6 male was mutagenized with ENU and bred to produce G1 founder males (59, 60). G1 males were crossed with C57BL/6J females to create the G2 generation. Recessive mutations were found in the G3 generation by a backcross of G2 females with their G1 father. The candidate Mpi mutation (p.H54R) was knocked in using sgRNA and single-stranded oligodeoxynucleotide (ssODN) (Supplemental Table 2). The *benadryl* allele was isolated by crossing first-generation CRISPR mice (C1) to C57BL6/N mice, then intercrossing the second-generation CRISPR mice (C2) to test third-generation CRISPR mice (C3) with the homozygous p.H54R mutation. The *Mpi*^fl^ allele was created using sgRNA targeting introns 3 and 4 of *Mpi* with ssODN containing *loxP* sequences (Supplemental Tables 1 and 2). Albumin-Cre (The Jackson Laboratory stock 003574) (61), Myh11-CreER^T2^ (Myh11-ERT2-Cre, The Jackson Laboratory stock 019079) (62), *Villin*-Cre (Vil1-Cre, The Jackson Laboratory stock 004586) (63), and CD45 (The Jackson Laboratory stock 002014) mice were obtained from The Jackson Laboratory. Mice 6 weeks to 12 months of age were used with age- and sex-matched littermates used as controls unless otherwise described.

### Western blot.

Tissues were lysed by sonication in NP-40 lysis buffer with protease inhibitors, normalized to equivalent total protein levels by Bradford Protein Assay Kit, and separated by electrophoresis (NuPAGE 4%–12% Tris gel). Antibodies from Santa Cruz Biotechnology (E-4), Boster Bio (11I4), and MyBioSource (4G9-B4-B8) were used to detect MPI from HEK293T cell lysate (ATCC). The 4G9-B4-B8 antibody clone was determined to produce optimal results and was used for reported experiments on murine tissues.

### qPCR.

TRIzol extraction (Thermo Fisher Scientific) of murine tissue in the QIAGEN PowerLyzer yielded pure total RNA used for qPCR experiments. cDNA was produced with random hexamers per the manufacturer’s protocol (Takara, PrimeScript 1st Strand cDNA Synthesis Kit). Predesigned primers for *Mpi* exons 4–5 were purchased from Integrated DNA Technologies (Mm.PT.58.6004801). We used 500 μM forward and reverse primers with SYBR Green Master Mix. GAPDH PrimeTime qPCR primers (Mm.PT.39a.1, IDT) were used to calculate the ΔΔCt value of *Mpi* in mutant and WT samples.

### Enzyme assay.

We used a modified protocol based on previously described methods (26, 58). Briefly, RBCs were lysed and the remaining WBCs were sonicated in 50 mM HEPES with a protease inhibitor cocktail (Cell Signaling Technology). Total protein levels were normalized after quantitation with the Bradford Protein Assay Kit (Pierce). Reaction components included 50 μg protein, 100 U phosphoglucose isomerase (yeast extract, MilliporeSigma), 500 mU glucose-6-phosphate dehydrogenase (MilliporeSigma), 5 μM MgCl_2_, and 1 mM NADP^+^. The reaction solution was preincubated to 37°C in an Agilent BioTek Synergy HTX plate reader, and M6P 0.03–8 mM was added to start the reaction. NADPH production was monitored by absorbance at 345 nm. Background was subtracted using the protein-only control, and each time point was adjusted by using the 0 mM M6P control. The first 3 minutes of data points were discarded, and then the slope of the linear portion of the reaction was used to calculate V_0_. GraphPad Prism 9 was used to plot V_0_ versus M6P and to calculate the *K_m_* and V_Max_.

### Glycopeptide mass spectrometry.

Glycopeptide mass spectrometry was performed by the UTSW Proteomics Core as described previously (65). Our method for determining N-glycan site unoccupancy was as follows: When a glycopeptide was found with no N-glycan attached, it was called unoccupied; when glycans were present, they were structurally named and classified as occupied. The total amount of glycopeptide detected was added together, and the unoccupied amount was divided by the total.

### DEXA scan.

Adipose, lean soft tissue, and fluid weights of mice were measured by MRI (66). Levels were normalized to total BW.

### Hematology analysis.

Complete blood count was measured from blood anticoagulated with EDTA by the HemaVet 950FS (Drew Scientific), which provided RBC count, size characteristics, and WBC count with differential. Reticulocytes were measured by staining blood cells with thiazole orange (1 ng/mL for 30 minutes) and detecting thiazole orange and anti-CD71 (allophycocyanin, RI7217, BioLegend) (42).

### BM transplant.

BM was acquired from the tibias and femurs of donor mice as described previously and processed as briefly described below (43). One to 5 million BM cells were transplanted (intravenous retro-orbital injection) into lethally irradiated recipient mice (total of 12 Gy irradiation given in 2 doses over 6 hours). Recipient mice were maintained on water treated with antibiotics (trimethoprim/sulfamethoxazole) from day –1 to +30 from transplant. Complete blood count was determined 8 weeks after transplantation to ensure engraftment.

### Serum biochemical measurements.

Several analytes were measured in nonfasting animals in accordance with commercially available ELISA kits, including ATIII (Biotang Inc.), insulin (Ultra Sensitive Mouse Insulin ELISA Kit, Crystal Chem), and IGF-1 (Invitrogen). Other serum analytes (AST, ALT, albumin, total protein, and iron) were measured by the VITROS 350 System in the UTSW Metabolic Phenotyping Core. Glucose homeostasis was measured by fasting the mice (14 hours overnight without food), then testing capillary blood glucose (AlphaTRAK Glucose Monitor, Abbott Animal Health) (66, 67). An i.p. glucose tolerance test was performed using 1 mg/kg glucose and measuring capillary blood glucose for up to 2 hours.

### PLE: A1AT in stool.

To assess PLE, feces were collected in individually housed mice for 4–6 hours. The feces were dried, weighed, and lysed in NP-40 buffer with protease inhibitors (1 mL/100 μg, end-to-end rotation at 4°C for 48 hours). The mixture was centrifuged (8,000*g* for 10 minutes), supernatant was collected, and A1AT was measured by ELISA (A1AT ELISA Kit, MyBioSource, and mouse-specific A1AT SimpleStep ELISA Kit, Abcam, ab267809) (68). The presence of Hgb in stool was further assessed by spotting 10 μL of supernatant on Hemoccult SENSA slides with developing agent (HemoCue); the sample would appear blue if hemoglobin were present.

### Histology.

Tissues were fixed in 4% paraformaldehyde for at least 48 hours before being paraffin-embedded. They were cut at 5 μm sections and stained with H&E, trichrome, PAS, or Alcian blue by the histology core facility.

### Mannose supplementation.

To rescue mice from the phenotypes associated with *Mpi* deficiency, we placed mice on drinking water with 2% mannose (The Vitamin Shoppe) and monitored their total BW, serum albumin levels, and blood counts over 30 days.

### Statistics.

Statistics were used for 2-group comparisons (Student’s 2-tailed *t* test for parametric data and χ^2^ and Mann-Whitney *U* test for nonparametric data) and 3 or more group comparisons (1-way ANOVA with Tukey’s multiple comparisons test). For 2 groups with multiple time points measured, we used 2-way ANOVA with Bonferroni’s correction, and for 3 or more groups with multiple time points measured, we used a mixed effects model with Tukey’s multiple comparisons test. A *P* value less than 0.05 was considered significant.

### Study approval.

All animal experiments were performed in accordance with the UTSW Medical Center IACUC–approved protocols.

### Data availability.

Data are available in the main article or the Supporting Data Values file.

## Author contributions

JAS and BB designed the research studies. JAS and BB acquired data. EBL, ZZ, and JMM analyzed the data. EBL, JAS, SM, TA, ZZ, AR, JMM, and WJI conducted experiments. JAS and BB were in charge of project administration and providing reagents. JAS and BB gathered resources. JQ, SH, SL, ENG, ZZ, and JR performed validation. JAS wrote the original draft. JAS, BB, SM, ZZ, and EBL reviewed and edited the manuscript.

## Supplementary Material

Supplemental data

Unedited blot and gel images

Supporting data values

## Version 1 07/22/2025

Electronic publication

## Version 2 07/24/2025

The graphical abstract was added
