# Supplementary material for: A hypomorphic Mpi mutation unlocks an in vivo tool for studying global N-glycosylation deficiency
Source: JCI Insight. 2025 Jul 22;10(14):e180752. doi: 10.1172/jci.insight.180752 (PMC12288971; doi:10.1172/jci.insight.180752)
Supplement: Supplemental data [file jciinsight-10-180752-s284.pdf]

Supplementary Material for: A hypomorphic *Mpi* mutation unlocks an in vivo tool for studying global *N*-glycosylation deficiency

Authors: Elisa B. Lin[1], Steve Meregini[1], Zhao Zhang[2, 3], Avishek Roy[1], Tandav Argula[1], James Mitchell[1], William J. Israelsen[4], Sara Ludwig[3], Jamie Russell[3], Jiexia Quan[3], Sara Hildebrand[3], Evan Nair-Gill[2], Bruce Beutler[3], Jeffrey A. SoRelle[1,3,5]*

Affiliations:

1. Department of Pathology, University of Texas Southwestern Medical Center, Dallas, TX, USA
2. Division of Endocrinology, Department of Internal Medicine, University of Texas Southwestern Medical Center, Dallas, TX, USA
3. Center for Genetics of Host Defense, University of Texas Southwestern Medical Center, Dallas, TX, USA
4. Department of Biochemistry, University of Texas Southwestern Medical Center, Dallas, TX, USA
5. Division of Allergy/Immunology, Department of Pediatrics, University of Texas Southwestern Medical Center, Dallas, TX, USA

*Corresponding Author
Jeffrey A. SoRelle, M.D.
Department of Pathology
University of Texas Southwestern Medical Center
5323 Harry Hines Boulevard
Dallas, TX 75390
Office: +1 214-648-4111
Email: Jeffrey.SoRelle@utsouthwestern.edu

Figure S1

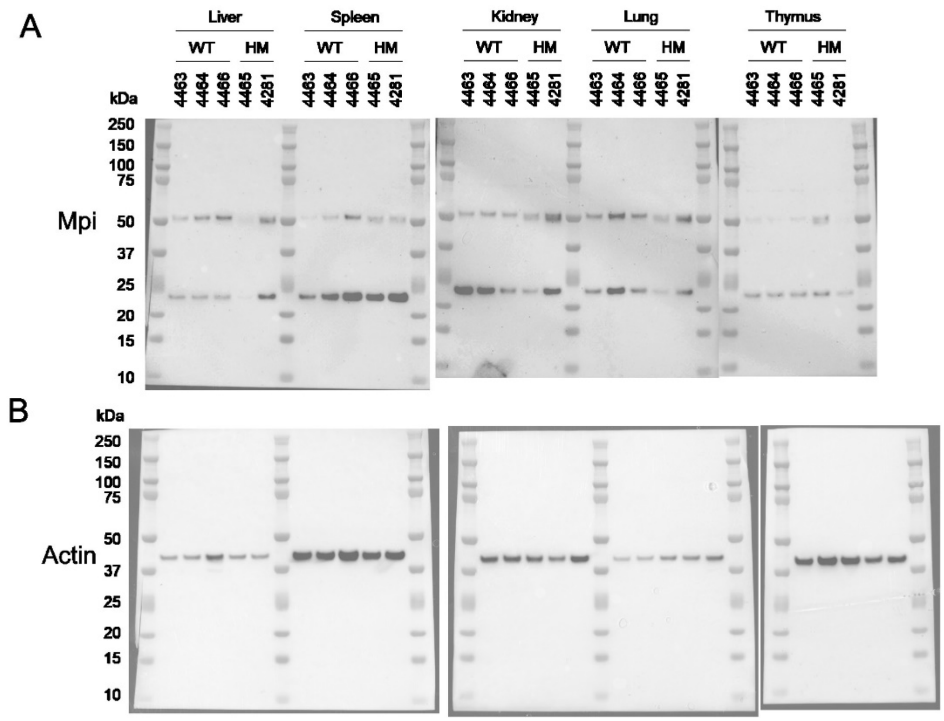

Supplemental Figure 1. **Extended Mpi protein expression.** Western blot of liver, spleen, lung and thymus for A) Mpi and B) Actin.

Figure S2

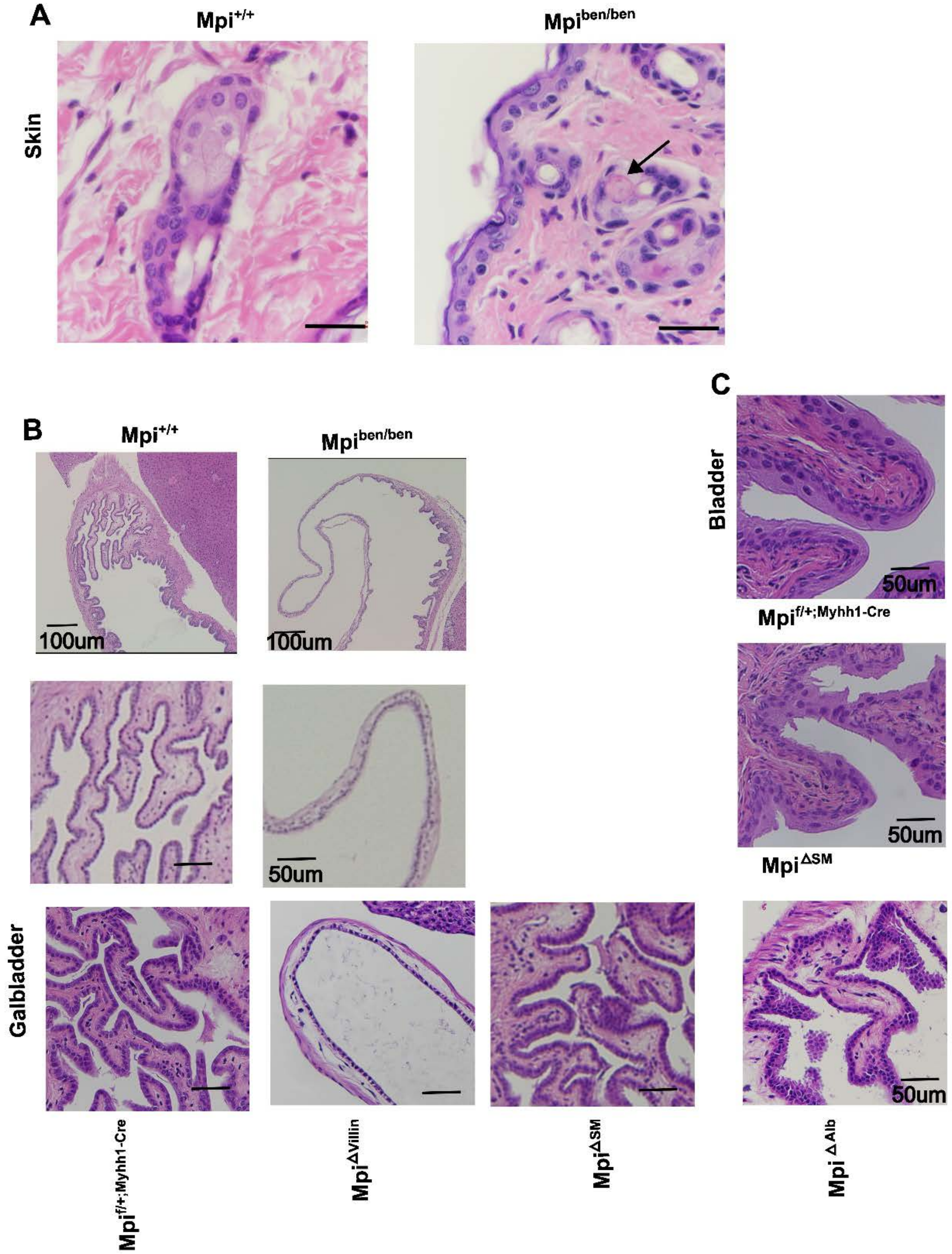

Supplemental Figure 2. **Histopathology of bladder and gallbladder**. (**A**) Representative microscopic images of hematoxylin and eosin (H&E)-stained sections of skin with focus on sebaceous glands and inclusions (arrow), and (B) gallbladders from *Mpi*$^{f/+}$; *Myh11*$^{ERT2\text{-}Cre}$, *Mpi*$^{f/f}$; *Villin*^{Cre} (*Mpi*$^{\Delta Villin}$), *Mpi*$^{f/f}$;*Myh11*$^{ERT2\text{-}Cre}$ (*Mpi*$^{\Delta SM}$), *Mpi*$^{f/f}$;*Alb*^{Cre} (*Mpi*$^{\Delta Alb}$), *Mpi*$^{+/+}$, and *Mpi*$^{ben/ben}$ mice. (**B**) Representative microscopic images of hematoxylin and eosin (H&E)-stained sections of bladders from *Mpi*$^{f/+}$;*Myh11*$^{ERT2\text{-}Cre}$ and *Mpi*$^{f/f}$;*Myh11*$^{ERT2\text{-}Cre}$ mice.

Figure S3

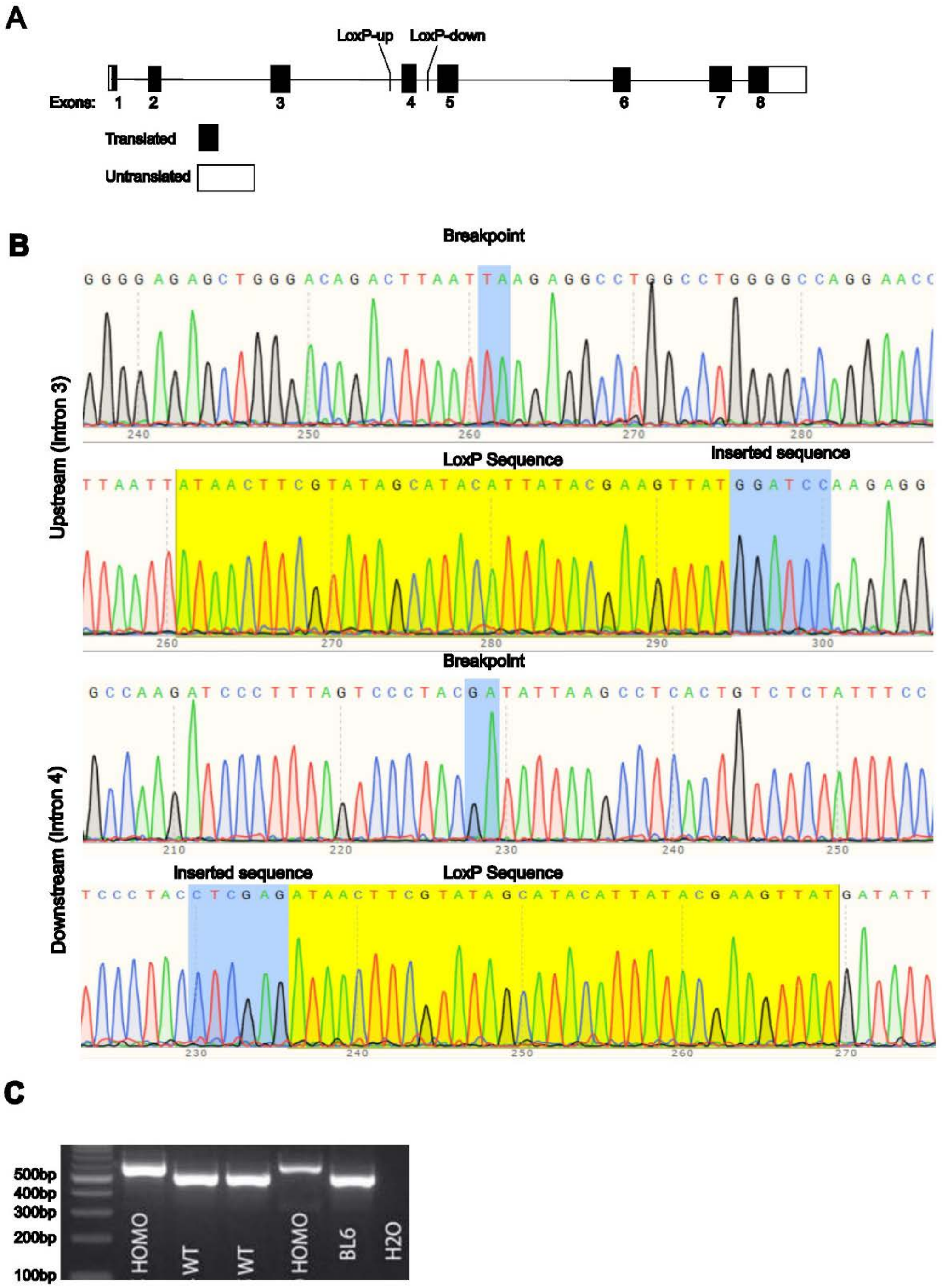

Supplemental Figure 3. **Diagram of *Mpi*^{flox} allele targeting and confirmation by sequencing and gel electrophoresis.** (**A**) Location of CRISPR gRNA targets for *Mpi*^{flox} alleles. (**B**) Capillary gel electrophoresis sequencing results demonstrating insertion of loxP sequences (highlighted in yellow) in the upstream (top) and downstream (bottom) locations. (**C**) Results of PCR and gel electrophoresis from primers flanking the two loxP sites. WT gene product is ~500 bp in length, while the *Mpi*^{flox} allele is 73 bp longer, at ~573 bp.

Figure S4

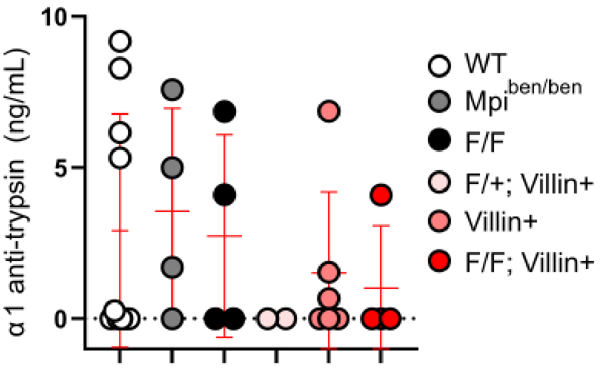

Supplemental Figure 4. **Assessing protein-losing enteropathy in stool**. Alpha-1 antitrypsin was measured in stool pellets collected from mice housed individually in cardboard boxes for 30-60 minutes. The stool was mixed with lysis buffer and was sonicated and centrifuged; the supernatant was used in an ELISA for *Mpi*$^{+/+}$, *Mpi*$^{ben/ben}$, *Mpi*$^{f/f}$, *Mpi*$^{f/+}$;*Villin*^{Cre}, *Villin*^{Cre}, and *Mpi*$^{f/f}$;*Villin*^{Cre} mice (n = 8, 4, 4, 2, 6, 4). Data points represent individual mice. Error bars indicate SD. Data represent values from two experiments combined. Most values were below the limit of detection.

Figure S5

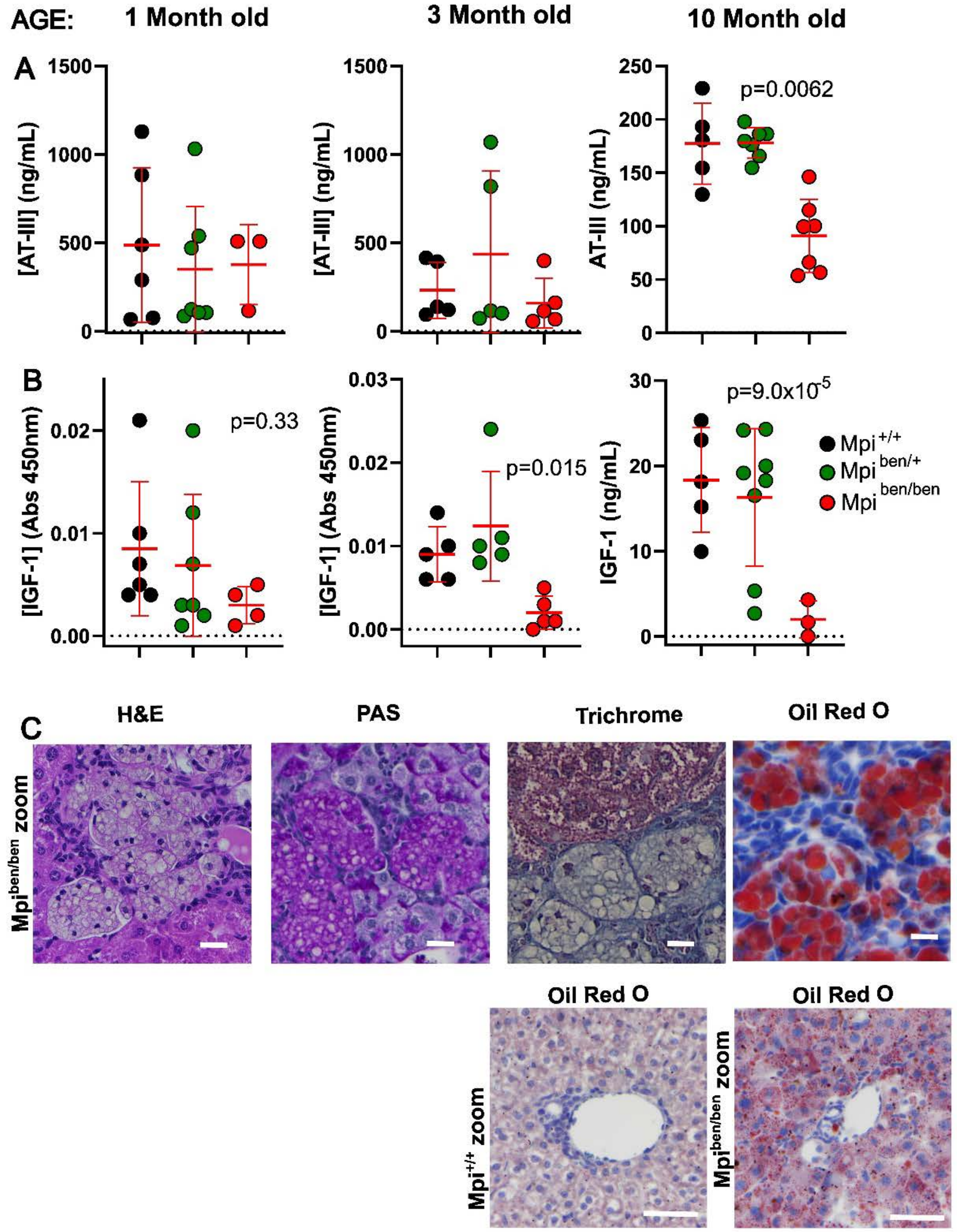

Supplemental Figure 5. **Antithrombin III and IGF-1 levels by age.** (**A**) Antithrombin III (AT III) in serum was measured by ELISA in *Mpi*$^{+/+}$, *Mpi*$^{ben/+}$, and *Mpi*$^{ben/ben}$ mice at one month (n = 6, 7, 3) and three months of age (n = 5, 5, 5), and ten months of age (n = 5, 7, 7). (**B**) Insulin-like growth factor 1 (IGF-1) in serum was measured by ELISA in *Mpi*$^{+/+}$, *Mpi*$^{ben/+}$, and *Mpi*$^{ben/ben}$ mice at one month (n = 6, 7, 4), three months of age (*n* = 5, 5, 5), and ten months of age (*n* = 5, 8, 3). Data points represent individual mice. Error bars indicate SD. *P*-values were determined by CRISPR *P*-value calculator for a recessive mode of inheritance. Lines in histology images

represent 25 μm. (**C**) Zoomed in focus on the macrophage (top row) aggregates in the livers of Mpi$^{ben/ben}$ mice stained with hematoxylin and eosin, periodic acid Schiff, trichrome, or Oil Red O. The bottom row focuses on wild type and Mpi$^{ben/ben}$ hepatocytes stained by Oil Red O.

Supplemental Table 1. Tracking of Mendelian ratios of *Mpi benadryl* and KO allele breedings.

Supplemental Table 1			
Het x Het Breeding (*benadryl)*			
**Genotype**	**Number weaned**	**Number expected**	
WT	30	30	100%
Het Replacement	61	60	102%
Homo Replacement	18	60	60%
Het-KO x Het Breeding (*benadryl)*			
**Genotype**	**Number weaned**	**Number expected**	
WT	17	13	131%
Het Replacement	15	13	115%
Het KO	19	13	146%
Compound Het	0	12	0%

Supplemental Table 2. CRISPR vectors used for targeting *Mpi* and creating *benadryl* replacement allele.

Supplemental Table 2	
benadryl CRISPR system	Alt-R® CRISPR-Cas9 crRNA, 2 nmol
crRNA_*Mpi*	TGGATGGGGACACACCCCCG
ssODN_*benadryl*	gatacatgcttcatacatccagggctgtcagttgcttcctttttctcaactttcctgttcttc cagctgtggatggggacaCGCccccggggagatgccaagatccttgacaacc gtatttcccagaagaccttaggccagtggattgctgaaaacccggactgcttgggct caaaggtcaaaaacacctttaatggaa
Flox CRISPR system	Alt-R® CRISPR-Cas9 crRNA, 2 nmol
**crRNA_LoxP_*Mpi*_Up_T**	CTGGGACAGACTTAATTAAG
**crRNA_LoxP_*Mpi*_Down_T**	CAGTGAGGCTTAATATCGTA
Product	4 nmole Ultramer® DNA Oligo
**ssODN_UpLoxP-*Mpi*_T**	atctaactctacctcccctaaatcatttaccctcatcacacagcttatgtgcctcctgtt tagggttcctggccccaggccagg**cctcttATAACTTCGTATAATGTA TGCTATACGAAGTTATCTCGAGaattaagtctgtcccag**ctctccc ctggcccatgttt
**ssODN_Down-LoxP-*Mpi*_T**	cctcagcaaccacacctgagcctggtctcaccctctggcccactggcccctaagac tgcctcatgaggccaagatccctttagtccctacCTCGAGATAACTTCGT ATAGCATACATTATACGAAGTTATgatattaagcctcactgtctctattt cccctctggccc
