# Supplementary material for: A hypomorphic Mpi mutation unlocks an in vivo tool for studying global N-glycosylation deficiency
Source: JCI Insight. 2025 Jul 22;10(14):e180752. doi: 10.1172/jci.insight.180752 (PMC12288971; doi:10.1172/jci.insight.180752)

Unedited Blots for: A hypomorphic *Mpi* mutation unlocks an in vivo tool for studying global *N*-glycosylation deficiency

Authors: Elisa B. Lin[1], Steve Meregini[1], Zhao Zhang[2, 3], Avishek Roy[1], Tandav Argula[1], James Mitchell[1], William J. Israelsen[4], Sara Ludwig[3], Jamie Russell[3], Jiexia Quan[3], Sara Hildebrand[3], Evan Nair-Gill[2], Bruce Beutler[3], Jeffrey A. SoRelle[1,3,5]*

Affiliations:
Department of Pathology, University of Texas Southwestern Medical Center, Dallas, TX, USA
Division of Endocrinology, Department of Internal Medicine, University of Texas Southwestern Medical Center, Dallas, TX, USA
Center for Genetics of Host Defense, University of Texas Southwestern Medical Center, Dallas, TX, USA
Department of Biochemistry, University of Texas Southwestern Medical Center, Dallas, TX, USA
Division of Allergy/Immunology, Department of Pediatrics, University of Texas Southwestern Medical Center, Dallas, TX, USA

Figure 2B

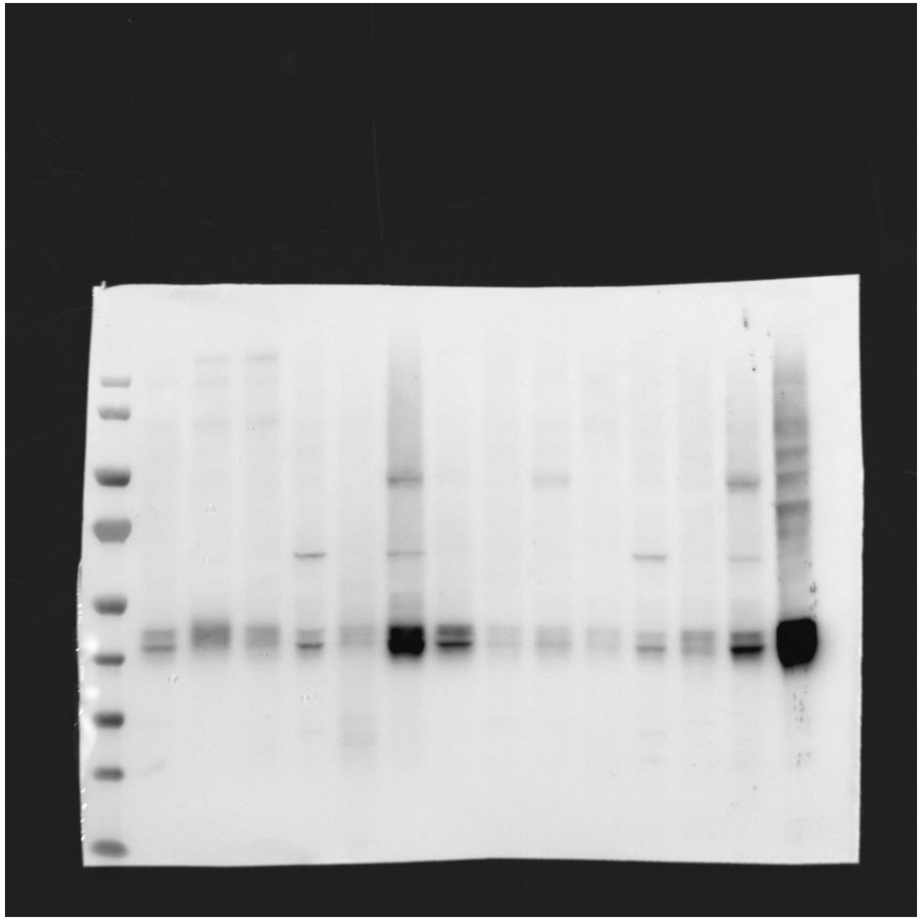

Figure S1 (full unedited included in Supplement

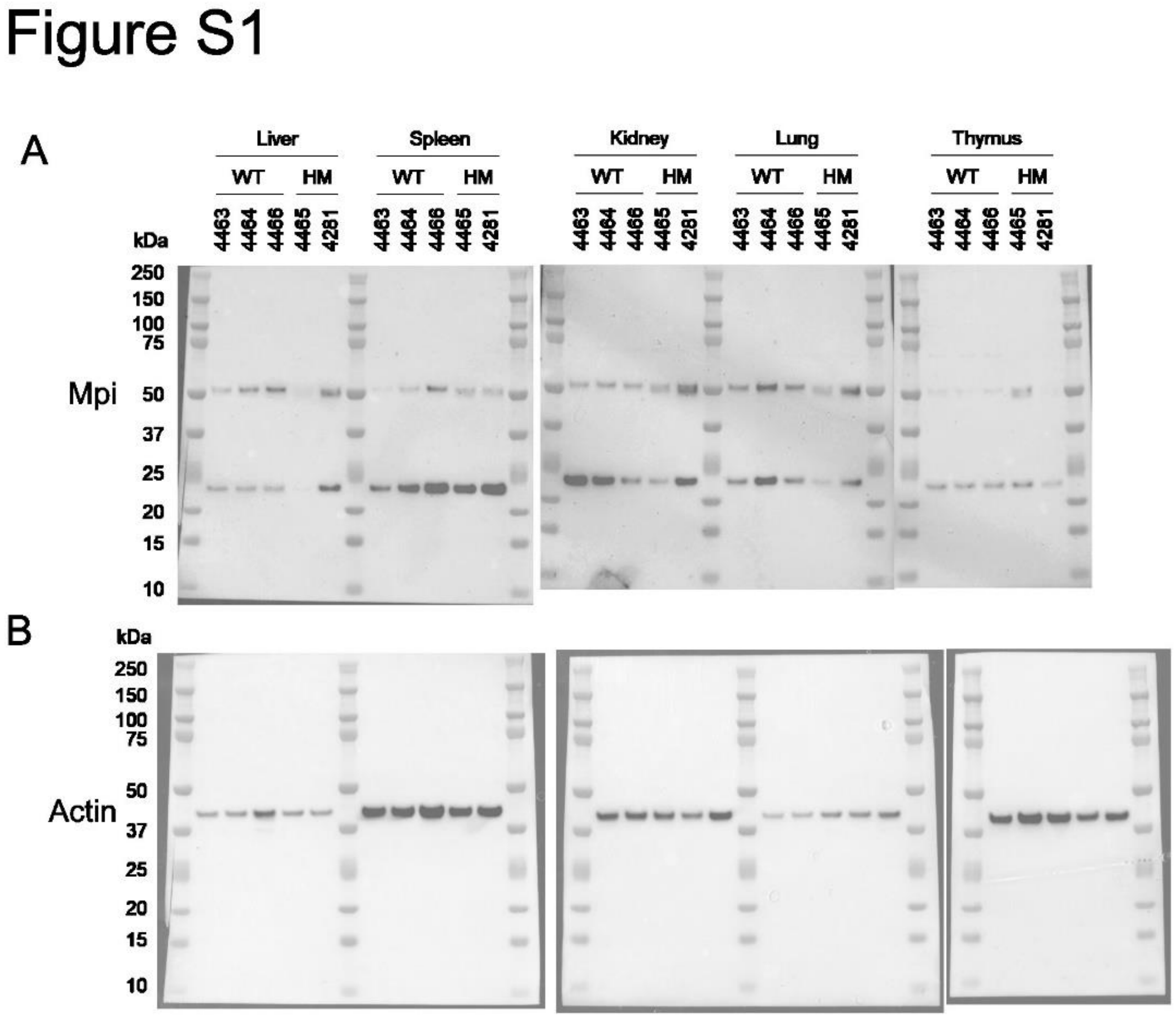

Supplement: Unedited blot and gel images [file jciinsight-10-180752-s285.pdf]
